# Photothermal therapy triggering organelle stress and metabolic reprogramming to potentiate antitumor immunity

**DOI:** 10.3389/fimmu.2026.1890839

**Published:** 2026-07-31

**Authors:** Lin Chen, Hanyu Zhang, Yunlong Wang, Xianhu Feng, Yi Hou, Qiang Su

**Affiliations:** 1 Nanchong Key Laboratory of Individualized Drug Therapy, Department of Pharmacy, Beijing Anzhen Nanchong Hospital Capital Medical University & Nanchong Central Hospital, The Second Clinical Medical College, North Sichuan Medical College, Nanchong, Sichuan, China; 2 Clinical Pharmacy and Pharmacy Department of The People’s Hospital of Zhongjiang, Deyang, Sichuan, China

**Keywords:** immunometabolism, metabolic reprogramming, organelle stress, photothermal therapy, tumor microenvironment

## Abstract

Metabolic reprogramming is a hallmark of malignant tumors, providing tumor cells with energy and promoting immune escape through changes in glucose, lipid, and amino acid metabolism. Photothermal therapy (PTT) not only eliminates tumor cells through localized hyperthermia but also disrupts metabolic networks. However, the mechanisms by which PTT-induced metabolic perturbation engages antitumor immunity remain a considerable challenge. This review proposes that PTT interferes with tumor metabolism through organelle stress and synergizes with exogenous drugs to enhance metabolic perturbation, thereby eliciting a potent antitumor immune response. We first detail how hyperthermia and ROS induced by PTT damage organelles, leading to organelle stress, including mitochondrial depolarization, endoplasmic reticulum (ER) proteotoxicity, cytosolic enzyme denaturation, and nucleolar stress. Critically, stressed organelles release immunogenic signals, activating the cyclic GMP-AMP synthase (cGAS)-stimulator of interferon genes (STING) pathway to trigger innate immune recognition, and regulating the functions of CD8^+^ T cells and macrophages through metabolites in the tumor microenvironment (TME). We also discuss how tumor cells activate adaptive responses, such as heat shock protein (HSP) upregulation and metabolic switching, to resist sublethal thermal stress, and evaluate synergistic strategies designed to amplify organelle stress and overcome thermotolerance. Finally, we focus on the latest advancements in overcoming tumor heterogeneity and advancing the clinical translation of targeted organelle PTT nanoplatforms. It is expected to elucidate the mechanism by which organelle stress in PTT causes metabolic perturbation, the tumor adaptive response, and its impact on tumor immunity, providing innovative ideas and references for the development of metabolic and tumor combined treatment strategies based on PTT.

## Graphical Abstract

**Figure:**
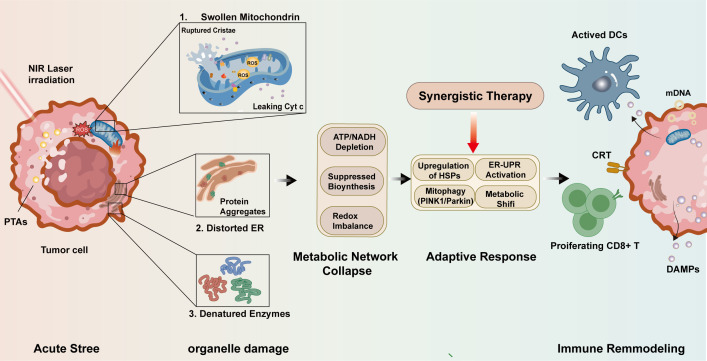

## 1 Introduction

Cellular metabolism is a series of enzymatic reactions that convert nutrients into energy, biosynthetic precursors, and reducing equivalents. Eukaryotic cells adjust their energy requirements by altering metabolic mechanisms to either enhance or reduce biosynthetic reactions, thereby resisting external environmental stress and enabling new cellular functions (1). This phenomenon is particularly prominent during tumorigenesis. Cancer cells autonomously alter various metabolic pathways to meet the increased demands for biological energy and biosynthesis, and to alleviate the oxidative stress associated with required for cancer cell proliferation and survival (2). For example, aggressive tumors such as triple-negative breast cancer (TNBC) and pancreatic ductal adenocarcinoma exhibit profound disruptions in glycolysis, lipid synthesis, and glutamine metabolism, which are consistently associated with poor prognosis (3, 4). Metastatic cells further enhance their metabolic dependencies on ATP production to sustain survival and colonization within hostile microenvironments (5). Furthermore, aberrant metabolism shapes an immunosuppressive TME by depleting nutrients such as glucose and tryptophan while accumulating lactate. These metabolic shifts collectively impair the function of cytotoxic T lymphocytes (CTLs) (6). Emerging evidence also implicates metabolic enzymes in epigenetic remodeling and T-cell exhaustion, emphasizing the importance of metabolism in immune escape (7). While targeting metabolic pathways presents a potential therapeutic strategy, single-agent treatments frequently fail due to compensatory adaptations (8). This finding highlights the urgent need for integrated strategies that concurrently target multiple metabolic vulnerabilities.

Photothermal therapy (PTT) has recently gained attention as a spatially precise and minimally invasive technology for cancer treatment (9). Photothermal agents (PTAs) are utilized to convert near-infrared (NIR) light into heat, selectively raising the local tumor temperature to within the range of 40°C–45°C. This localized hyperthermia effectively induces cell death while sparing surrounding healthy tissues (10). PTT has shown promise in both preclinical and initial clinical trials for treating superficial cancers such as breast cancer (BC) and skin cancer (11, 12). A variety of PTAs, including organic dyes, polymeric nanoparticles, noble metals, and carbon-based materials, offer versatile platforms for targeted delivery and functional integration (10, 13). Conventionally, PTT efficacy is attributed to heat-induced protein denaturation, membrane disruption, and DNA damage (14). Recent findings indicate that PTT also affects tumor metabolism, causing rapid mitochondrial depolarization, which suppresses oxidative phosphorylation (OXPHOS), depletes ATP, and triggers reactive oxygen species (ROS) bursts (14–16). Moreover, PTT disrupts glucose and nucleotide metabolism, impairing DNA repair and synergizing with thermal damage (17). Interventions targeting amino acid and lipid metabolism, such as inhibition of fatty acid synthase (FASN), can further suppress metastatic potential (18, 19). Changes in metabolite release, including lactate and damage-associated molecular patterns (DAMPs), collectively reprogram the metabolism of immune cells within the TME. For instance, PTT has been shown to promote the glycolytic metabolism of CD8^+^ T cells and trigger macrophage proinflammatory polarization, reversing the immunosuppressive TME (20). Metabolomic studies reveal significant metabolic changes following PTT, including alterations in glutamate, uridine nucleotides, glycerophosphocholine, and phenylalanine derivatives, further supporting the rationale for targeting tumor metabolism through PTT (21, 22).

In this review, we propose that in addition to the direct thermal damage to the plasma membrane and cytoskeleton, the organelle stress induced by PTT also affects tumor metabolism by disrupting mitochondrial, ER, and cytoplasmic homeostasis. The organelle stress, combined with exogenous drugs, ions, or genetic interventions, can amplify the metabolic perturbation induced by PTT. This metabolic perturbation alters metabolites such as lactate and ATP in the TME, thereby reshaping the tumor immune microenvironment. This article mainly focuses on the association between PTT-induced organelle stress and immune outcomes, rather than systematically exploring the mechanisms of plasma membrane rupture or cytoskeleton degradation. Instead, we position organelle stress as a central hub through which PTT-based combination therapies achieve enhanced antitumor efficacy. We first summarize the organelle stress triggered by PTT-induced heat and ROS. We then discuss how these stresses, together with changes in the metabolite profile, influence infiltrating immune effector cells, including CD8^+^ T cells, macrophages, and natural killer (NK) cells. Finally, we evaluate tumor heterogeneity and the translational challenges, including batch-to-batch reproducibility, biodegradability, nontargeted heating, laser penetration depth, and regulatory complexity.

## 2 PTT-induced metabolic disruption via multiorganelle damage

**Figure 1:**
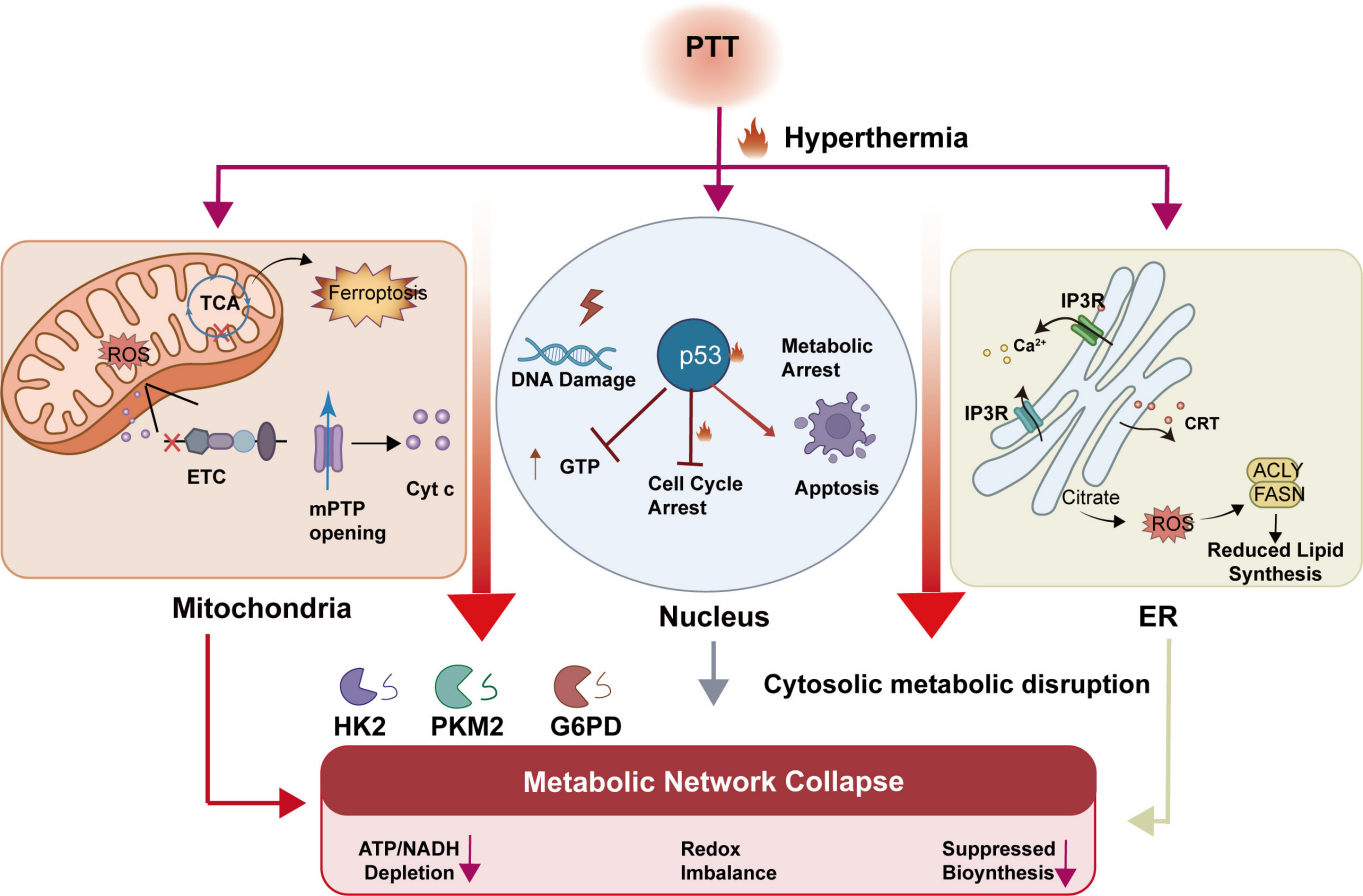
Acute disruption of tumor metabolic networks by PTT-induced multiorganelle stress. PTT induces localized hyperthermia and a ROS burst, impairing the functions of the mitochondria, the ER, and the nucleus. The stress disrupts metabolism, leading to ATP/NADH exhaustion, inhibition of biosynthesis, and ultimately to metabolic dysfunction, promoting apoptosis and ferroptosis.

This section examines how PTT disrupts tumor metabolism by inducing multiorganelle damage. We detail the impairment of the mitochondria, the ER, the cytosol, and the nucleus, resulting in acute metabolic collapse (**Figure 1**). Although this review draws on conventional hyperthermia studies for clues, PTT exhibits different biophysical effects (23).

### 2.1 Distinct biological outcomes of conventional thermal ablation versus photothermal therapy

Conventional hyperthermia typically heats the large volume of tissue where the tumor is located to 39 °C–45°C using an external heat source. As an adjunctive therapy, it enhances tumor blood perfusion to improve chemotherapeutic drug delivery and sensitizes cancer cells to radio- and chemotherapy (24). In contrast, thermal ablation is performed under image guidance, such as ultrasound or CT, by percutaneously inserting a probe into the tumor and generating localized temperatures exceeding 60°C to induce instantaneous coagulative necrosis of tumor cells (25). Distinct from these probe-dependent modalities, PTT utilizes PTAs to generate localized and precise heating within the tumor, avoiding the side effects caused by excessive thermal damage. When exposed to laser light, PTAs absorb light energy through localized surface plasmon resonance (SPR) at the nanoscale and convert the light energy into heat through nonradiative relaxation. Therefore, the heat generation can be spatially confined, enabling highly targeted heating of biological tissues. Research has found that the temperature changes in tumor cells during PTT occur in two stages: a transient stage and a steady state stage. The temperature rises rapidly from zero to the peak, with a time constant (τ) of approximately 1.4–12.9 ns, and depends on the particle size and tissue type (26). These ultra-fast thermal shock mechanisms for damaging cancer cells, such as protein denaturation and membrane rupture, are completely different from those of conventional hyperthermia. In the steady-state stage, the temperature of PTT is characterized by a saturated temperature δT_max_, and the temperature remains stable while reaching the maximum value during the continuous laser activation period.

Traditional PTT typically raises the local temperature above 45°C, leading to necrosis of tumor cells (27). When the temperature exceeds 50°C, the tertiary structure of proteins is denatured, DNA double-strand breaks occur, and enzymatic activities are abolished, resulting in the immediate collapse of metabolic networks (28). Moreover, excessive heat disrupts the structural integrity of tumor-associated antigens (TAAs) and DAMPs, thereby eliminating their immunoadjuvant properties (29). The concomitant vascular destruction further prevents immune cell infiltration, thereby reducing the effectiveness of traditional PTT in immunotherapy (29, 30). In contrast, mild photothermal therapy (mPTT) (42 °C–45°C) induces sublethal thermal stress without complete metabolic arrest (31). When glycolysis and OXPHOS are inhibited, tumor cells can activate tumor adaptive responses, thereby weakening the photothermal therapeutic effect. Notably, this mitochondrial damage kills tumor cells by inhibiting the function of the antiapoptotic proteins Bcl-2/Bcl-xl and increasing the expression of the proapoptotic protein BAX (32). The dead tumor cells release TAAs and DAMPs, thereby forming an *in situ* vaccine to activate antigen-presenting cells (APCs). Furthermore, mPTT promotes vasodilation, increases blood perfusion, disrupts the dense extracellular matrix, and reduces interstitial fluid pressure, thereby overcoming physical barriers and facilitating the infiltration of effector immune cells, such as T cells and NK cells, and chimeric antigen receptor (CAR) T cells (33).

### 2.2 Mitochondrial stress and dysfunction

The cytotoxic effect of PTT is attributed to its physical disruption of subcellular structures and organelles. Studies have found that mitochondrial parameters such as oxygen consumption and mitochondrial membrane potential (MMP) are significantly decreased in tumors treated with PTT (34). The functional integrity of mitochondria is mainly compromised by protein denaturation, membrane damage, and oxidative stress caused by hyperthermia (35).

**Figure 2:**
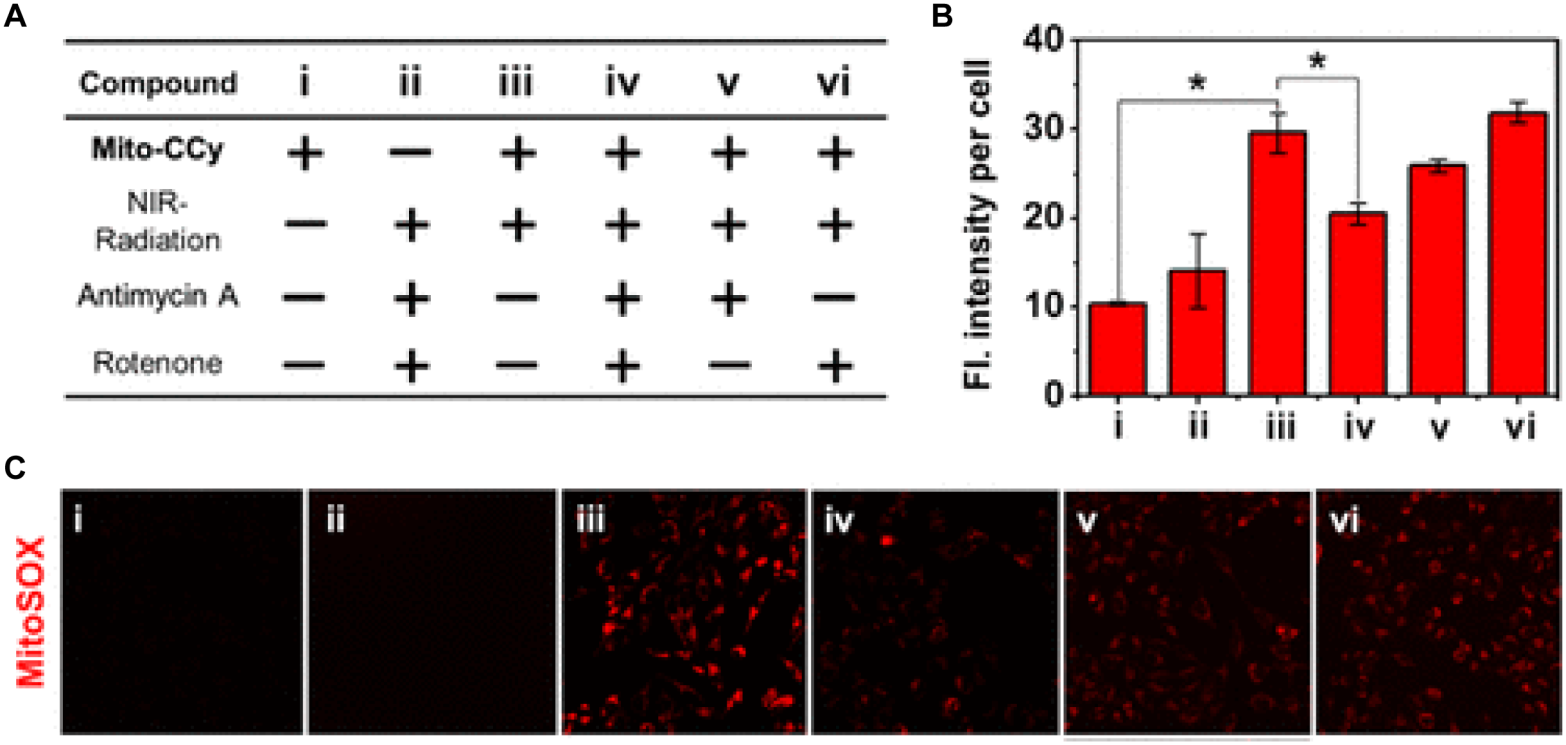
Use of a ROS indicator to evaluate the production of ROS in mitochondria after incubation of HeLa cells with MitoSOX. **(A)** Matrix showing the experimental design. **(B)** Fluorescence intensity per cell; ^*^p < 0.05. **(C)** Confocal fluorescence microscopic images of HeLa cells treated with MitoSOX (1.0 μM) according to the treatment variables, including coincubation with Mito-CCy (10 μM), with or without 730 nm NIR irradiation (2.3 W cm^−2^) for 10 min, antimycin A (0.5 μM), and rotenone (0.5 μM). Fluorescence images were recorded using an excitation wavelength of 488 nm and an emission wavelength of 550−650 nm (red). A Ti: Sa femtosecond-pulsed laser (Chameleon XR by Coherent, 200 fs pulse width, 90 MHz repetition rate) was used. The energy density (fluence) was 1,380 J cm^−2^ (23). Copyright 2017, ACS.

Studies have found that conventional mild hyperthermia (40°C) increases the levels of ROS in tumor cells, including superoxide and H_2_O_2_. Under these conditions, ROS levels are closely related to mitochondrial membrane hyperpolarization (36, 37). Recent research further indicates that PTT disrupts the electron transport chain (ETC), which is a primary step in triggering endogenous ROS generation in mitochondria (23) (**Figure 2**). In this experiment, mitochondrial complexes I and III were confirmed to be involved in the production of mitochondrial endogenous ROS. According to the paradigm established by Murphy, mitochondrial O_2_·^−^ production is critically governed by the proton motive force (Δp) and the reduction state of the coenzyme Q (CoQ) pool (38). We infer that the thermal stress caused by PTT will increase Δp and simultaneously over-reduce the CoQ pool. This thermodynamic state may promote the reverse electron transfer (RET) of CoQH_2_ to complex I and stabilize the ubisemiquinone at the Qo site of complex III, thereby generating endogenous O_2_·^−^ (39, 40). However, this experiment only focused on complexes I and III, leaving the potential contribution of complex II unexplored. This is particularly relevant as complex II directly interfaces with the CoQ pool and generates O_2_·^−^ at high rates during both forward and reverse electron transfer, especially under other physiological stimuli (41). The dysfunction of the ETC causes a significant increase in electron leakage, leading to O_2_·^−^ generation (42). Meanwhile, ROS-mediated cardiolipin peroxidation impairs respiratory chain complexes III and IV, potentially inhibiting the production of ROS by the ETC (43).

Furthermore, the redox systems within cells are also closely related to ROS. Numerous studies have shown that conventional hyperthermia also triggers a decline in mitochondrial antioxidant capacity, further promoting the generation of ROS (44). Szukalska et al. found that SOD activity in the kidneys of mice in the laser irradiation group was significantly reduced by approximately 20% (45). However, Jung et al. demonstrated that the heat generated by PTT is unlikely to inactivate SOD or other antioxidant enzymes in mitochondria to mediate the effect (23). These contradictory findings are resolved by considering the distinct temporal windows of observation. Jung et al. examined acute effects within minutes after irradiation, ruling out direct thermal inactivation of SOD. In contrast, Szukalska et al. measured renal SOD activity at day 40 posttreatment, a time point reflecting chronic metabolic toxicity and systemic redox depletion rather than acute heat damage. Thus, these observations are complementary rather than conflicting, delineating the transition from acute photothermal stress to long-term metabolic remodeling. Nevertheless, changes in antioxidant enzyme activities following PTT can influence downstream redox reactions beyond direct thermal inactivation. The O_2_·^−^ generated by mitochondrial ETC dysfunction interacts with cellular redox systems. For example, NO scavenges radicals and inhibits lipid peroxidation, but it rapidly reacts with O_2_·^−^ to form ONOO^−^ (46). The rate of ONOO^−^ formation depends critically on SOD activity, which regulates the level of O_2_·^−^ (47). A decrease in SOD activity induced by PTT, as observed in the kidneys and livers of mice treated with laser irradiation, may therefore promote ONOO^−^ accumulation, exacerbating oxidative damage and altering antioxidant defenses (45).

Multiple studies have shown that the endogenous ROS and heat produced by PTT jointly cause mitochondrial dysfunction and cell apoptosis (23, 48). When exposed to laser irradiation, PTAs cause a decrease in mitochondrial membrane potential by inducing thermal damage, thereby confirming the phenomenon of mitochondrial depolarization (48). Therefore, this mitochondrial dysfunction inhibits OXPHOS in tumors. Additionally, ROS generated by PTT can trigger the continuous opening of the mitochondrial permeability transition pore (mPTP), thereby causing mitochondrial swelling and outer membrane rupture (49, 50). Moreover, ROS can cause mitochondrial impairment by intensifying oxidative stress within cells, consequently hindering ATP production (51).

### 2.3 ER stress and proteotoxicity

The ER plays a vital role in the synthesis, folding, modification, and quality control of proteins in tumor cells (52). PTT triggers extensive denaturation and misfolding of proteins within cells, leading to ER swelling and the accumulation of misfolded proteins in the ER lumen (53). This exceeds the folding and processing capacity of the organelle, thereby triggering endoplasmic reticulum stress (ERS) (54). Studies have confirmed that the expression of ERS markers, such as phosphorylated eukaryotic translation initiation factor 2α (eIF2α), C/EBP homologous protein (CHOP), and activating transcription factor 6 (ATF6), is upregulated after traditional hyperthermia (24, 55, 56). However, the inositol-requiring protein 1α (IRE1α)-X-box binding protein 1 (XBP1) pathway may be suppressed under PTT, as indicated by reduced XBP1 expression (54). This difference implies that the thermal effect and ROS burst caused by PTT may have exceeded the adaptive regulation of the IRE1α-XBP1 pathway, leading to its suppression. The research has found that NIR light (808 nm) mediated PTT can induce strong ROS-dependent ERS in the ER, characterized by the upregulation of CHOP expression (57). This ERS prompts the efficient translocation of CRT protein from the ER to the cell surface, stimulating the maturation of the dendritic cells (DCs) and enhancing the immune response. Compared with nontargeted systems, ER-targeted PTT can upregulate glucose-regulated protein 78 (GRP78) by approximately twofold (58).

ROS disrupt the redox environment within the ER and trigger the unfolded protein response (UPR) in synergy with hyperthermia during PTT (53). Studies have shown that ROS produced by PTT can inhibit the function of molecular chaperones and interfere with the formation of disulfide bonds (59). Concurrently, ROS induced by PTT can oxidize ER proteins and lipids, further exacerbating structural and functional damage. Certain nanomaterials used in PTT, such as gold nanorods or polymeric nanoparticles, further amplify this effect by increasing local ROS generation (60). For instance, treatment with positively charged BPEI-gold nanoparticles has been shown to disrupt cellular redox homeostasis and antioxidant response pathways, indicating a significant disturbance of ER homeostasis (60). This direct physical and oxidative damage serves as the initial trigger for subsequent ER stress responses.

Disruption of calcium homeostasis provides another example where findings from conventional hyperthermia studies inform our understanding of PTT-induced organelle stress. The ROS produced by PTT are also closely related to the imbalance of Ca^2+^. Studies have shown that ROS oxidatively modify calcium ion channels such as the ryanodine receptor 2 (RyR2) located on the ER membrane. Oxidative stress can lead to the unzipping of the domains between the N-terminal (1–220) and central (2,300–2,500) domains of RyR2, disrupting the stability of its tetramer structure and subsequently causing calmodulin (CaM) to dissociate from the channel (61). Traditional hyperthermia damages ER membrane integrity, disrupts calcium channels, and denatures ER-resident chaperone proteins such as GRP78/Binding immunoglobulin protein (BiP), thereby impairing protein folding capacity and calcium homeostasis. A study found that exposure of HeLa cells to hyperthermia (42 °C–43°C) for 3–24 h significantly increased cytoplasmic free calcium levels, which can activate the calcium-dependent proteolytic system, calpain-calpastatin (62). Interestingly, the cell heat tolerance induced by preheating at 40°C effectively maintains ER calcium homeostasis. This, in turn, inhibits the calcium release from the ER caused by lethal hyperthermia, thereby preventing cytoplasmic calcium overload and blocking the abnormal activation of calcium-dependent proteases (58). However, whether these findings apply directly to PTT is uncertain. Unlike conventional hyperthermia, PTT delivers millisecond-to-second thermal spikes and steep intracellular gradients (26). Such conditions may provoke acute calcium channel gating defects and ER membrane permeabilization through mechanisms distinct from those of gradual heating.

### 2.4 Cytosolic metabolic disruption

Beyond mitochondria, the cytosol is another primary target of PTT-induced damage. PTT disrupts glycolysis and the pentose phosphate pathway (PPP) through heat stress and ROS. The disruption of these metabolic pathways is primarily mediated by metabolic enzymes in the cytosol.

#### 2.4.1 Heat-induced denaturation and degradation of glycolytic enzymes

Traditional localized heating denatures glycolytic enzymes, such as hexokinase, phosphofructokinase, and pyruvate kinase, impairing their catalytic activity and slowing glycolytic flux (63). In addition to the ubiquitin-mediated degradation caused by conventional hyperthermia, studies have demonstrated that enzymes can undergo direct thermal denaturation under photothermal conditions. Thompson et al. demonstrated that by coupling horseradish peroxidase (HRP) and glucose oxidase (GOx) with gold nanorods, selective inactivation could be achieved within 90 s of near-infrared light irradiation through local plasmonic heating (64). This effect is attributed to the extreme temperature gradient near the surface of the nanorods. This principle supports the view that the heat generated by PTT can directly impair the function of enzymes. However, whether this applies to specific glycolytic enzymes in the complex environment of cells still requires further research. Most existing studies have not focused on directly verifying this thermal inhibition. On the contrary, metabolic enzymes are often used as sensitive targets and are combined with chemotherapy drugs to enhance the efficacy of PTT. These studies may demonstrate that the hyperthermia generated by PTT has a limited inhibitory effect on metabolic enzymes.

#### 2.4.2 ROS-mediated dysfunction and metabolic inhibition in the cytosol

Beyond ROS generated directly by PTT, cytosolic NADPH oxidase represents another potential source of ROS (65). A study indicated that conventional hyperthermia triggers an increase in NADPH oxidase activity in U-2 OS cells in a temperature-dependent manner, increasing the generation of O_2_·^−^ (66). ROS generated by PTT can oxidize the protein thiol groups of complexes I, II, IV, and V (F_0_F_1_-ATPase), causing carbonylation of glycolytic enzymes (67). Specifically, heat stress inhibits the mitochondrial localization of hexokinase 2 (HK2) via ROS, reducing its binding to voltage-dependent anion channel (VDAC) and suppressing glycolysis (68). This effect may be particularly significant in tumors relying on mitochondrial-bound HK2 for survival. However, there is a lack of evidence to link the changes in enzyme subcellular localization caused by PTT with the real-time decrease in glycolytic flux. Meanwhile, the ROS produced by PTT can upregulate the protein expression of glucose-6-phosphate dehydrogenase (G6PD), enhancing the phosphoribosylation cycle of the tumor. ROS also activate poly (ADP-ribose) polymerase, which consumes NAD^+^ and further compromises ATP production (65). This forms a vicious cycle in which DNA repair consumes NAD^+^, thereby increasing energy depletion. The energy demand of PTT further exacerbates cytosolic ATP depletion. The synthesis and function of HSPs are highly ATP-dependent (69). The massive ATP consumption by HSP expression, coupled with the inhibition of glycolytic enzymes, such as phosphoglycerate kinase and pyruvate kinase, results in an irreversible energy crisis (70).

### 2.5 Nuclear stress and p53-mediated metabolic reprogramming

The heat generated by PTT is a physical stimulus. How this stimulus is transduced into biochemical signals within the cell nucleus remains controversial. Some studies suggest that under mild hyperthermia (42 °C–45°C), nuclear stress is induced by cytoplasmic stress signals, such as ROS generation and calcium ion influx (71). In contrast, other studies have observed rapid physical rupture of the nuclear membrane at higher temperatures (72). Therefore, whether nuclear stress is a direct target or an indirect consequence of PTT may vary under different treatments.

#### 2.5.1 Nucleolar stress and energy metabolism

PTT triggers stress responses within the cell nucleus, such as DNA damage and nucleolar stress. The nucleolus is the site of ribosome biosynthesis and is crucial for protein synthesis, which supports the high energy demands of tumor proliferation (73). Additionally, the nucleolus has been identified as a stress sensor that integrates stress signals (74). In response to photothermal stimulation, tumor cells activate nucleolar stress responses, resulting in inhibited ribosome biosynthesis, cell cycle arrest, or apoptosis. The typical characteristics include changes in nucleolar morphology and the translocation of nucleolar proteins from the nucleolus to the nucleoplasm. Studies have shown that mPTT using laser irradiation of tumor cells significantly increases GTP levels, promotes ribosome synthesis, and leads to the enlargement of the nucleolus (22). However, normal MCF-10A cells showed no significant changes in nucleoli during irradiation, and no obvious apoptosis was observed. These findings confirm that tumor cells are more sensitive to thermal stimulation than normal cells. In the early stage, there is a rapid increase in ATP and GTP synthesis, potentially providing energy substrates for the expression of HSPs and stress repair. In the later stage, the levels of nucleotides sharply decline, accompanied by the collapse of mitochondrial membrane potential and cell membrane rupture, eventually leading to apoptosis. The accumulation of GTP within the nucleus is more significant than that of ATP, suggesting that it may have a special role in the stress signal transduction of the nucleolus (22). Another study indicates that the intranuclear phosphorylated protein nucleophosmin 1 is transferred from the nucleus to the cytoplasm, activating the TPL2–NPM–Mdm2–p53 pathway, aligning with the nucleophosmin 1 (NPM1) translocation phenomenon observed during mPTT (74, 75). After 12 min of irradiation, nucleoli underwent fragmentation and complete loss of characteristic features, at which point apoptosis occurred. Unstable nucleoli can release ARF and various ribosomal proteins (RPs) into the cytoplasm, which then bind and inhibit the E3 ligase activity of Mdm2, resulting in the stabilization and activation of the p53 pathway (76–78). The severity of this nucleolar stress response is further amplified by the unique thermal microenvironment of the perinuclear region. Gao et al. revealed that the thermal conductivity near the nucleus can be as low as 0.035 W m^−1^ K^−1^, which would severely restrict heat dissipation from this organelle-rich zone and thereby exacerbate thermal-induced nucleolar protein unfolding and release (79). Thus, these observations also prove that PTT-induced nucleolar stress activates the p53 pathway to intervene in the metabolic processes of tumor cells. For example, activated p53 suppresses glycolysis by upregulating TIGAR and downregulating glycolytic enzymes or transporters, such as LDHA, HK2, and glucose transporter 1 (GLUT1) (80–83). In some cases, p53 may promote the transition to oxidative metabolism, although this effect is cell type and stress intensity-dependent. For instance, p53 transcriptionally activates the synthesis of cytochrome *c* oxidase 2 (SCO2), a crucial assembly factor for complex IV of the ETC, thereby promoting OXPHOS (84). Additionally, p53 upregulates carnitine *O*-octanoyltransferase (CROT), which converts very-long-chain fatty acids into medium-chain fatty acids for mitochondrial β-oxidation (85). Beyond its role in energy metabolism, p53 suppresses *de novo* purine synthesis by inhibiting MTHFD2, thereby limiting nucleotide availability for DNA repair (86). These understandings reveal the pivotal role of p53 in connecting the DNA damage response with cellular metabolism.

#### 2.5.2 DNA damage and p53-mediated metabolic reprogramming

When the local temperature exceeds 50°C, such rapid heating breaks the hydrogen bonds and denatures the DNA double helix, causing immediate double-strand breaks (87). PTT-induced rapid heating causes proteins to unfold and denature. Protein aggregation and denaturation have significant effects in the cell nucleus, which contains a high concentration of proteins and DNA (88). At an ablation temperature of 48 °C–60°C, proteins undergo extensive and irreversible denaturation (88). However, molecular chaperones such as HSP70 can facilitate reversibility under mild PTT (89). Therefore, the mPTT range (42 °C–45°C) is not sufficient to induce substantial DNA damage. More importantly, excessive heat can denature proteins, alter the structure of enzymes, and lead to the inactivation of enzymes responsible for DNA repair, such as those involved in homologous recombination or nonhomologous end joining (87). Proteomics studies have found that hyperthermia combined with chemotherapy can lead to a large number of differentially expressed proteins, with nearly two-thirds related to DNA damage repair in heat-sensitive ovarian cancer cells. The DNA repair-associated protein retinoblastoma-like protein 1 shows a marked increase following thermochemotherapy, potentially enhancing chemosensitivity due to hyperthermia (90). Whether PTT alone, without chemotherapeutic agents, can similarly upregulate retinoblastoma-like protein 1 (RBL1/p107) remains unclear. Notably, p53 pathway activation induced by thermochemotherapy, through stabilization of the p53 protein and degradation of its negative regulators such as JMJD5, reprograms tumor metabolism (91). However, whether mPTT alone triggers p53 activation through similar kinetics remains to be directly tested.

## 3 Tumor adaptive responses

**Figure 3:**
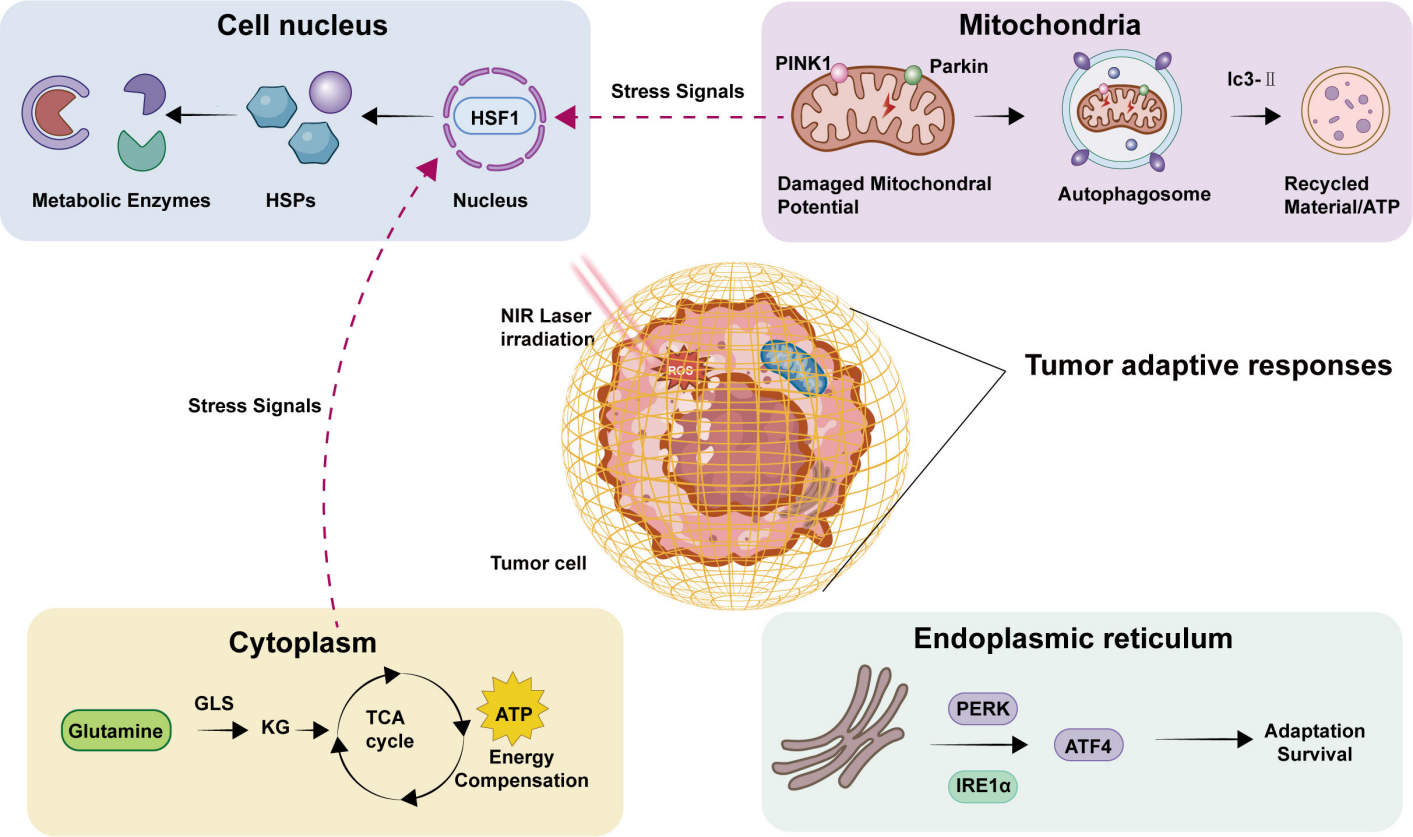
Tumor adaptive mechanisms and thermoresistance in response to PTT-induced stress. Tumor cells respond to PTT-induced thermal and oxidative stress by activating multiple compensatory programs. These include the upregulation of HSPs, the recycling of damaged mitochondria through mitophagy, ERS, and metabolic reprogramming that enhances glutamine utilization to sustain bioenergetics.

In response to PTT-induced proteotoxic, oxidative, and metabolic stress, tumor cells rapidly activate adaptive responses, including stress signaling, chaperone upregulation, organelle remodeling, and metabolic reprogramming. While initially protective, these responses can ultimately compromise PTT efficacy by promoting cell survival and triggering resistance. This section summarizes the adaptive mechanisms, including the heat shock response, ERS, mitochondrial quality control, and metabolic plasticity under PTT (**Figure 3**).

### 3.1 HSPs and thermotolerance

The direct damage caused by PTT to the mitochondria, ER, and cytoplasm induces extensive protein denaturation, misfolding, and ROS bursts, thereby activating heat shock factor 1 (HSF1) in the cytoplasm. A study has shown that protein denaturation and aggregation caused by hyperthermia activate HSF1, leading to the upregulation of HSPs. At the same time, HSF1 also initiates the UPR in the ER to enhance the protein-folding ability of the ER. HSF1 also promotes the translation of mRNA for prosurvival proteins such as ATF4, providing the raw materials for the synthesis of HSPs (59). Additionally, PTT-induced mitochondrial dysfunction and ROS bursts further promote HSF1 activation through redox-sensitive signaling pathways (92). For instance, in the human monocytic cell line U937, H_2_O_2_ can activate HSF1 in a concentration-dependent manner (93). The ability of HSF1 to sense H_2_O_2_ and heat stress depends on two specific cysteine residues (Cys35 and Cys105) within its DNA-binding domain. A study has found that H_2_O_2_ may also impair the DNA-binding activity of HSF1 by oxidizing the cysteine residues within its DNA-binding domain (93). However, no direct evidence indicates that the H_2_O_2_ produced by PTT enhances the therapeutic effect by oxidizing the DNA-binding domain of HSF1. Furthermore, ROS can activate specific kinase pathways, such as the c-Jun N-terminal kinase (JNK) signaling pathway, which then phosphorylates and activates HSF1 (94). The activated HSF1 enters the nucleus and binds to the specific nGAAn pentamer repetitive DNA sequence within the heat shock element, thereby promoting the expression of HSP genes (69, 95). Among the numerous HSPs, HSP70 and HSP90 are the main members that function as molecular chaperones in the cytoplasm, relying on ATP. The efficacy of low-temperature PTT is mainly limited by the overexpression of HSP70 and HSP90 (96). They can utilize the energy provided by ATP hydrolysis to promote the native folding of their substrates, thereby preventing aggregation and correcting protein folding under stress (96). Studies have found that in thermotolerant cells exposed to 40 °C mild hyperthermia, the absence of HSP72 exacerbates ERS and counteracts the protective effect. Experimental results showed an increase in the phosphorylation levels of PKR-like endoplasmic reticulum kinase (PERK) (Thr980), eIF2α (Ser51), and IRE1α (Ser724), as well as an increase in sXBP1 expression. Additionally, HSP72 knockdown (KD) causes the thermotolerant KD cells to be sensitive to the activation of caspase 4, 7, and 3 (58). However, as mentioned earlier, the thermal effects and ROS produced by PTT damage these metabolic enzymes. Thus, PTT not only induces metabolic stress but also disrupts the compensatory mechanisms, driving cells toward death. Notably, the HSPs induced by PTT mediate tumor reprogramming by stabilizing and activating key proteins involved in metabolic regulation. For instance, in bladder epithelial cells, HSP90 interacts with PKM2 to prevent its proteasomal degradation, maintaining glycolytic flux to support cell proliferation (97). Additionally, HSP90 can stabilize the transcription factor c-MYC and subsequently activate the expression of downstream metabolic genes such as lactate dehydrogenase A (LDHA), promoting glycolytic metabolism (98). Therefore, the upregulation of HSPs caused by PTT provides protection for tumor cells to maintain their high metabolic state under stress, thereby providing a rationale for combining PTT with HSP inhibitors.

The HSP60 located in mitochondria is also one of the HSPs that reduce the efficacy of PTT in tumor treatment (99). Research indicates that AuNR-P17 can reduce the thermotolerance of tumor cells by downregulating HSP60 (100). HSP60 regulates the metabolic functions of tumor cells through its subcellular localization. Unlike HSP60 in tumors, HSP60 located on the cell membrane binds to integrin α3β1, promoting the metastasis of BC cells (101). This study demonstrates that disrupting the binding of HSP60 to integrin α3β1 can inhibit the metastasis of BC. Furthermore, HSP60 in mitochondria is closely related to tumor metabolism. For instance, in multiple myeloma, HSP60 is significantly upregulated and supports tumor proliferation by maintaining mitochondrial function (102). Knocking down HSP60 reduces the levels of metabolites in both glycolysis and the tricarboxylic acid (TCA) cycle, and affects the PPP, resulting in blocked synthesis of ribonucleotides and NADPH. Additionally, HSP60 can regulate the activity of metabolic enzymes such as PFKFB3 by modulating the adenosine monophosphate-activated protein kinase (AMPK) signaling pathway, thereby controlling fatty acid metabolism (102). Therefore, inhibiting HSP60 may enhance the efficacy of PTT by targeting tumor metabolism.

### 3.2 ERS and metabolic reprogramming

The UPR is rapidly activated as an adaptive response. The initial purpose of the UPR is to restore ER homeostasis by inhibiting global protein synthesis and upregulating the expression of molecular chaperones. The UPR is mainly regulated by three transmembrane proteins in the ER: PERK, IRE1α, and ATF6 (103, 104). Among them, the pathway triggered by PERK-mediated phosphorylation of eIF2α plays a crucial role in the adaptation to hypoxia and the generation of therapeutic resistance (105). PERK inhibits global protein translation by phosphorylating eIF2α and promotes the selective translation of ATF4, thereby upregulating the expression of ER chaperone proteins such as BiP. On the other hand, the IRE1α/XBP1 signaling branch can enhance the ER folding capacity synergistically manner to mitigate ERS (106). Studies have shown that HSP72 KD in thermotolerant cells exacerbates ERS upon mild thermal shock at 40°C, as evidenced by increased phosphorylation levels of PERK (Thr980), eIF2α (Ser51), and IRE1α (Ser724), as well as the upregulation of sXBP1 expression (58). However, the ERS induced by PTT is usually prolonged and severe, often shifting the UPR signaling toward proapoptotic pathways, such as the CHOP pathway (56). For instance, significant induction of pJNK could be observed in cells treated with PTT (53). The challenge lies in precisely controlling the stress intensity to ensure that PTT induces cell death rather than activating survival and thermotolerance mechanisms. Some research suggests that tumor cells may enhance survival by activating survival signaling pathways such as ERK and AKT in suboptimal PTT doses, which weakens the efficacy of PTT (53). On the other hand, intense ERS combined with autophagosome accumulation can collectively induce cell death (107). For instance, studies have observed the simultaneous presence of swollen ER and autophagosomes within cells after PTT, suggesting the coordination between the UPR and autophagy (53). The dual role of ERS in cell survival versus death is governed by the UPR signal strength and duration. Mild ERS activates adaptive PERK-eIF2α signaling to restore ER homeostasis, whereas severe ERS triggers PERK–CHOP and IRE1α–JNK proapoptotic cascades. Bettaieb et al. demonstrated that mild preconditioning at 40°C for 3 h induces Hsp72, which physically interacts with the cytosolic domain of IRE1α to enhance prosurvival XBP1 signaling while restraining PERK–eIF2α hyperphosphorylation, thereby maintaining ER homeostasis and establishing thermotolerance. In contrast, sustained exposure to 42–43°C for ≥ 2 h overwhelms this Hsp72 buffering capacity, leading to sustained eIF2α phosphorylation, CHOP accumulation, Ca^2+^-dependent calpain activation, and subsequent caspase-4/3-mediated apoptosis (58). Cell-type-specific susceptibility further determines the net outcome. For instance, basal ERS levels were significantly higher in the MCC type and cells with high MUC2 expression than in the NMCC type and cells with low MUC2 expression. The mucinous characteristics of these tumors (excessive secretion of MUC2 protein) were the main reason for the increase in basal ERS levels (108). Thus, PTT-induced ERS is a modifiable determinant rather than a fixed response, offering a rationale for the therapeutic fine-tuning required to shift stress from adaptive to lethal.

Notably, the ER induced by PTT may activate specific prosurvival pathways, thereby reducing the therapeutic effect. Studies have shown that PTT significantly upregulates the expression of TXNDC5, weakening the therapeutic effect by modulating ER function (109). This finding highlights that tumor cells can utilize the ER adaptive response induced by PTT to enhance their survival. To address this, Yin et al. constructed an NIR-II-responsive CRISPR/Cas9 system combined with two-dimensional (2D) nanomaterials (CRISPR/Cas9-2D) system combined with two-dimensional (2D) nanomaterials silicene nanosystem for the precise knockout of TXNDC5 (109). This intervention effectively remodeled ER function, reprogrammed metabolic pathways, and consequently potentiated PTT efficacy while inhibiting tumor recurrence.

### 3.3 Mitochondrial quality control and energy supply

In response to PTT-induced damage, tumor cells rapidly activate adaptive responses, including autophagy. Autophagy is a lysosome-dependent recycling process that degrades damaged organelles and proteins, thereby maintaining energy homeostasis and promoting cell survival (110). Under mPTT, autophagy promotes the removal of heat- and ROS-damaged mitochondria, maintaining intracellular homeostasis and potentially promoting cell survival (111). Mitophagy, a selective form of autophagy for mitochondria, can be triggered mainly via ubiquitin-dependent or receptor-mediated pathways. Growing evidence suggests that PTT preferentially activates the ubiquitin-dependent PINK1/Parkin pathway, which is composed of PTEN-induced putative kinase 1 (PINK1) and Parkin. PTT-induced localized hyperthermia and subsequent mitochondrial dysfunction lead to ROS accumulation. This oxidative stress promotes the expression of PINK1 and its stabilization on the OMM (112, 113). Once activated, PINK1 phosphorylates ubiquitin and recruits Parkin, an E3 ubiquitin ligase (114). Parkin then ubiquitinates mitochondrial outer membrane proteins, forming a signal recognized by autophagy receptors such as p62/SQSTM1. These receptors link the ubiquitinated mitochondria to microtubule-associated protein 1 light chain 3-II (LC3-II) on the forming autophagosome, thereby initiating mitophagy. This process is often coordinated with unc-51-like autophagy activating kinase 1 (ULK1) (115). Consistently, experimental studies have observed the upregulation of LC3-II and the downregulation of p62 following PTT, further confirming the activation of autophagy (116). Furthermore, during PINK1/Parkin-mediated mitophagy, tumor protein p53-induced nuclear protein 1 may reduce the generation of ROS induced by stress, which could impact Parkin recruitment and its E3 ligase activity (117). As a crucial prosurvival mechanism, PTT-induced autophagy not only recycles damaged components but also suppresses other cell death pathways. Enhanced autophagy can clear damaged organelles that might otherwise trigger pyroptosis, thereby promoting cancer cell survival (118). Conversely, inhibiting autophagy exacerbates organelle dysfunction and oxidative stress, leading to pyroptotic cell death (119). Therefore, inhibiting this prosurvival autophagy pathway can effectively reduce the thermotolerance of tumor cells, making them more sensitive to PTT. Inhibiting autophagy has been proven to enhance the effect of PTT (120). However, the outcome of PTT-induced mitophagy is not fixed. It is governed by the balance between autophagic flux capacity and mitochondrial damage burden. Mild stress can promote efficient clearance of damaged mitochondria via PINK1/Parkin, supporting cell survival; whereas severe stress inhibits this capacity, causing flux blockade and the accumulation of proapoptotic signals, such as cytochrome *c* and mitochondrial DNA (mtDNA), which induce cell death via the cGAS-STING and apoptotic pathways. Thus, autophagy inhibition sensitizes tumors to mPTT but may be detrimental at high PTT doses. Therefore, real-time autophagic flux monitoring, such as monitoring LC3-II/p62 dynamics, is warranted to guide PTT parameter selection for optimal therapeutic outcomes.

As another aspect of the prosurvival function, mitophagy induced by PTT can significantly alter cellular metabolism. Mitophagy can bidirectionally regulate the balance between glycolysis and OXPHOS. For instance, BNIP3L/NIX-dependent mitophagy promotes a shift toward glycolysis during retinal ganglion cell differentiation (121). Conversely, hypoxia-induced BNIP3-mediated mitophagy enhances OXPHOS while suppressing glycolysis in uveal melanoma (122). This metabolic reprogramming is often mediated by altering the pool of TCA cycle metabolites. In colon cancer, PINK1-mediated mitophagy significantly reduces acetyl-CoA levels through the p53 signaling pathway and the HIF-1α-PDHK1 axis, thereby suppressing tumor growth (123). This mitophagy-induced metabolic reprogramming is not limited to tumor cells. In cancer-associated fibroblasts (CAFs), tumor-derived exosomal ITGB4 protein can induce BNIP3L-dependent mitophagy and glycolysis, resulting in increased lactate production. These metabolically reprogrammed CAFs subsequently secrete factors that promote the proliferation, invasion, and metastasis of BC cells (124). This highlights that mitophagy inhibition may disrupt not only tumor cell-intrinsic survival but also the metabolic symbiosis within the TME. Therefore, the impact of PTT on tumor metabolic reprogramming must be considered along with the adaptive response triggered by mitochondrial autophagy.

### 3.4 Metabolic switch and metabolic flexibility

The thermal effect triggered by PTT, as a cellular stressor, elicits metabolic adaptation in tumor cells. On one hand, ATP is required for the synthesis of HSP70 and HSP90, which repair heat-damaged proteins (96). On the other hand, hyperthermia therapy may also temporarily modulate the metabolic reprogramming of tumor cells. Indeed, tumor cells exhibit different metabolic responses to heat stress. For instance, the mitochondrial function of heat-sensitive cells declines under intense heating, leading to an energy crisis (34). In contrast, thermotolerant cells may maintain ATP generation by reprogramming their metabolism. As a stressor, hyperthermia disrupts these metabolic fluxes, which are largely influenced by the specific metabolic pathway on which the cell line relies. A key factor in the variation of thermal responses is the functional capacity and adaptability of the mitochondrial respiratory chain. For instance, studies have shown that the same hyperthermia treatment increases the basal respiratory rate only in AGS gastric cancer cells, while having no such effect on Caco-2 colorectal cancer (CRC) cells and T3M4 pancreatic cancer cells (125). Kanamori et al. confirmed that hyperthermia can promote a metabolic shift from glycolysis toward OXPHOS. The ovarian cancer cell line SKOV3 compensates for the inhibition of glycolysis by activating mitochondrial OXPHOS, thereby maintaining ATP generation (34). The metabolites in the TCA cycle are speculated to be provided by glutaminolysis, which generates α-ketoglutarate. Future studies measuring glutamine flux are needed to verify this hypothesis. Therefore, glutamine metabolism may become an adaptive process under heat stress. However, this effect may be further inhibited by long-term exposure to hyperthermia. Current studies on the metabolic switch induced in tumor cells by PTT are still limited, and the mechanism has not been fully elucidated. Moreover, other regulators of the basal metabolic phenotype could be correlated with the switching of metabolic pathways. For instance, miR-373-3p targets MFN2 to promote glycolysis and alters the metabolic phenotype of tumor cells in CRC (126). Given that miR-373-3p has been shown to be regulated by other stresses such as hypoxia and to affect metabolism, future studies are warranted to explore its role under heat stress.

## 4 Disrupting multiorganelle functions to overcome tumor thermotolerance

It is important to emphasize that the strategies discussed in this section are predominantly combination-enhanced effects that require additional pharmacological, ionic, or genetic interventions beyond photothermal heating alone. The primary metabolic effects unequivocally attributable to PTT alone, including mitochondrial depolarization, ETC inhibition, ER stress, glycolytic enzyme inhibition, and HSF1 activation, have been detailed in **Section 2**. Here, we focus on how exogenous agents or genetic manipulations can be integrated with PTT to amplify organelle damage, disrupt compensatory metabolic pathways, and convert sublethal thermal injury into metabolic collapse. The following subsections summarize these strategies based on their primary organelle targets.

### 4.1 Targeting mitochondrial metabolism

Mitochondria are central to key metabolic processes, including OXPHOS, the TCA cycle, the synthesis of iron–sulfur clusters, and the regulation of cell death (127, 128). PTT can damage the MMP and the ETC. However, the combined strategy aims to exacerbate this damage through various mechanisms or to precisely exploit mitochondrial metabolic dependencies (14–16, 69).

#### 4.1.1 Interfering with mitochondrial ion homeostasis and electron transfer

**Figure 4:**
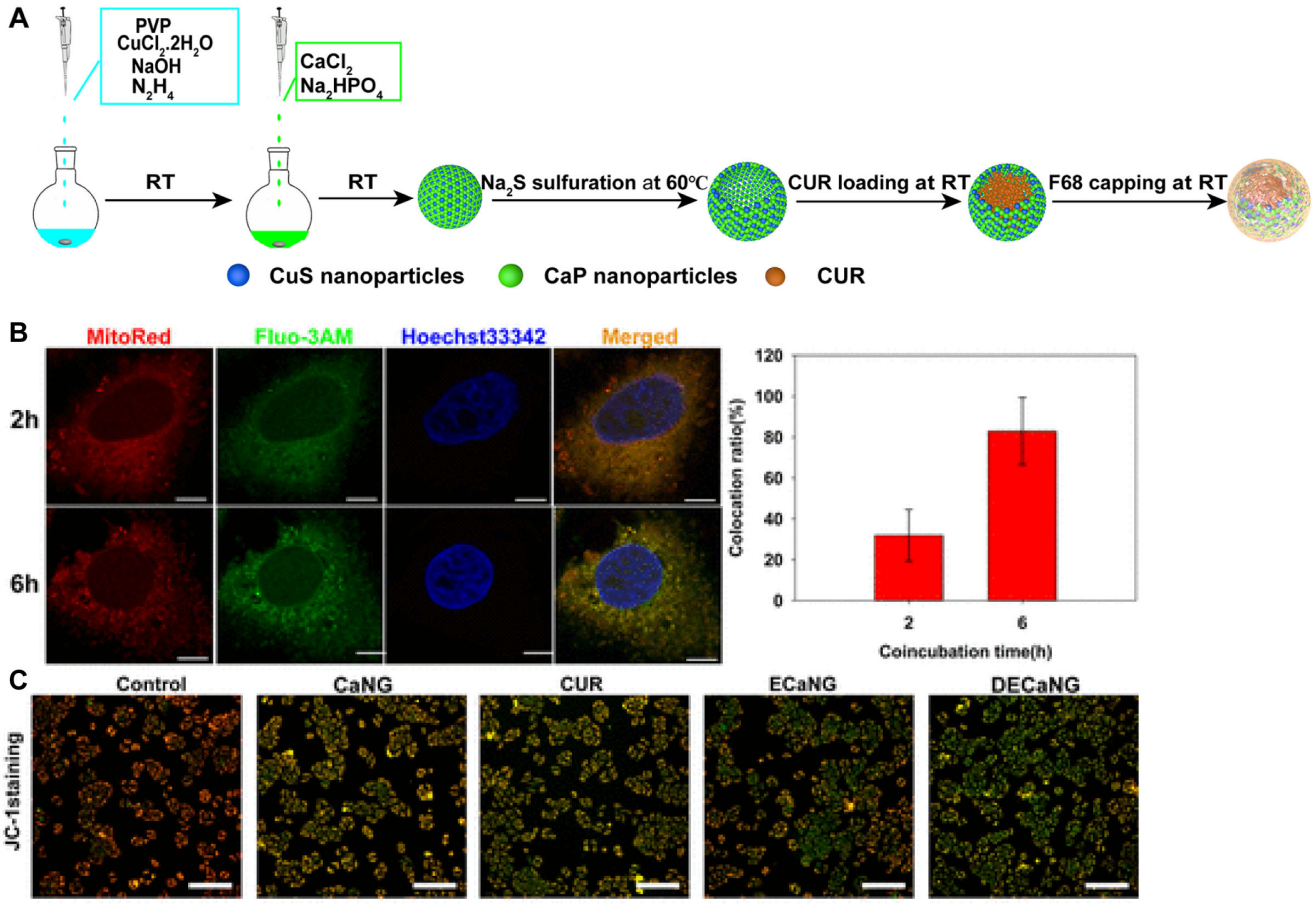
Mitochondrial Ca^2+^ overload and membrane potential disruption induced by ECaNG in MCF-7 cells. **(A)** Schematic illustration of the synthesis process of ECaNG. **(B)** CLSM images and colocalization ratio analysis of the biodistribution of Ca^2+^ released from CaNG in mitochondria after treatment with CaNG in MCF-7 cells for different times (*n* = 3). Scale bar: 7.5 μm. Mechanism study of cell apoptosis. **(C)** Mitochondrial membrane potential changes in MCF-7 cells treated with different preparations observed by fluorescence microscope. Scale bar: 100 μm (132). Copyright 2018, ACS.

Mitochondrial function is highly dependent on an intact MMP and ETC activity. The localized hyperthermia generated by PTT damages the integrity of the mitochondrial membrane, leading to the depolarization of the MMP (42). This hyperthermia inhibits the activity of ETC complexes, thereby blocking OXPHOS at the source. Further research has revealed that certain PTT nanomaterials, such as single-walled carbon nanotubes, can exacerbate mitochondrial respiratory dysfunction by disrupting the conformation and redox state of Cyt *c* (129). Based on this thermal damage, exogenous agents are designed to further disrupt mitochondrial ion homeostasis, inhibit matrix metabolic enzymes, or block outer-membrane transporter complexes, thereby exacerbating the energy crisis beyond the level achievable by PTT alone. The physical disruption of the mitochondrial membrane by PTT facilitates the invasion of exogenous ions. When PTT is combined with Zn^2+^-releasing nanoparticles, such as ZnO_2_@PDA, the exogenous Zn^2+^ further inhibits α-ketoglutarate dehydrogenase, thereby amplifying mitochondrial dysfunction beyond that induced by heat alone (70). The disruption of Ca^2+^ homeostasis represents another classical synergistic pathway. PTT can weaken the Ca^2+^ regulatory capacity of the ER and mitochondria (130, 131). The combined strategy, such as a DECaNG nanogenerator, damages mitochondria by delivering a high flux of Ca^2+^ into the mitochondria, thereby enhancing the efficacy of PTT (**Figure 4**). In coordination with PTT, this ionic storm induces the continuous opening of the mPTP, leading to the depolarization of the MMP and the release of Cyt *c* (132). The experiment further confirmed that Ca^2+^ might enhance PTT by downregulating the expression of HSP family genes. Studies have shown that activation of the Piezo1 ion channel can exacerbate heat-induced damage to cancer cells through the NOX2-ROS signaling pathway (133). This indicates that specific ion channels can be utilized to regulate the response to heat stress during PTT, which is closely linked to the pathological process of oxidative stress. Gas signal molecules provide another chemical intervention method for mitochondrial metabolism. NO binds to the heme center of ETC complex IV, exacerbating the respiratory depression caused by PTT at the molecular level (134). Similarly, hydrogen sulfide (H_2_S) has emerged as another target. Studies have shown that pharmacological downregulation of H_2_S levels can significantly delay tumor growth by inhibiting cellular respiration, ATP synthesis, and glycolysis, providing new insights for tumor treatment based on H_2_S clearance (135). Dong et al. combined H_2_S elimination with PTT to develop AZA-BOD-2 (136). The study results indicate that the controllable depletion of H_2_S, accompanied by photothermal ablation, significantly enhances the therapeutic efficacy for colorectal tumors while sparing surrounding healthy tissues in living mice.

#### 4.1.2 Targeting mitochondrial matrix metabolic enzymes and inducing cuproptosis

In addition to the ETC, another pivotal approach for combined treatment involves targeting metabolic enzymes located in the mitochondrial matrix. The most straightforward approach is to deliver metabolic inhibitors to the matrix to inhibit energy production. Specific inhibitors, such as ivosidenib, have been applied in clinical practice for tumors harboring mutations in TCA cycle enzymes, such as isocitrate dehydrogenase (IDH), succinate dehydrogenase (SDH), and fumarate hydratase (FH) (137, 138). These inhibitors not only disrupt tumor cell energy metabolism by blocking the TCA cycle, but also reduce the ATP level required for the cells to resist thermal stress (139). Combining these inhibitors with PTT represents a promising but underexplored strategy. Meanwhile, these inhibitors also reduce the production of lactate, laying the foundation for modulating the TME. The recently proposed concept of cuproptosis has provided a novel direction for synergistic PTT strategies. Copper ions bind to lipoylated enzymes of the TCA cycle in mitochondria, promoting their aggregation and inactivation, and triggering unique proteotoxic stress. For instance, MCD NPs promoted the release and targeted delivery of copper ions through hyperthermia, thereby synergistically achieving PTT and cuproptosis. The synergistic activation of this novel programmed cell death pathway has shown great potential independent of apoptosis and necrosis, especially in models such as osteosarcoma (140). In addition to the introduction of exogenous copper to trigger cuproptosis, Zhang et al. exploited the endogenous copper pool in tumor cells to activate a self-amplification cascade reaction, achieving highly efficient and low-toxicity cuproptosis therapy (141). This nanoparticle inhibited copper chaperones (ATOX1/CCS), leading to intracellular Cu^+^ accumulation and copper reduction, which was amplified by PTT. In addition, glutaminase 1 (GLS1) located in the mitochondrial matrix can also be targeted in combination with PTT to synergistically regulate glutamine metabolism. Inhibition of GLS1 blocks the energy replenishment derived from glutamine catabolism, causing thermal damage to tumor cells while they undergo energy depletion. Inhibition of GLS1 also disrupts GSH synthesis, weakening the intracellular antioxidant defense and impairing the clearance of ROS generated by PTT. For instance, the glutaminase inhibitor CB839 combined with PTT can further inhibit glutamine metabolism, leading to oxidative stress and ferroptosis (142). Studies have shown that the GLS1-mediated metabolic pathway is closely related to the expression and function of HSPs (143). Inhibiting GLS1 may interfere with the correct folding or the energy supply of HSPs, thereby increasing tumor cell sensitivity to PTT.

#### 4.1.3 Disrupt the transporter complexes on the OMM

In addition to targeting the internal mechanisms of mitochondria, the synergistic strategy with PTT also focuses on disrupting the pivotal transporter complexes on the OMM. The HK2 and VDAC complex on the OMM physically and functionally couples the first step of glycolysis to mitochondrial ATP, promoting tumorigenesis (144). For instance, T-3-BP-AuNPs (gold nanoparticles) targeting mitochondria not only suppress glycolytic flux by disrupting the VDAC complex but also promote Cyt *c* release, thereby simultaneously impairing energy production and inducing apoptosis (145). Similarly, the mitochondrial pyruvate carrier (MPC), which transports pyruvate into the mitochondrial matrix, can be inhibited to force cells to rely on inefficient glycolysis (146). This metabolic transformation, in synergy with the heat stress produced by PTT, consumes ATP and inhibits tumor growth. However, disruption of the VDAC complex also impacts mitochondrial calcium uptake, which is crucial for regulating metabolic enzymes and electrophysiological homeostasis in excitable cells (147). Therefore, this dual disruption can synergize with PTT to induce an energy crisis and cell death.

### 4.2 Proteotoxicity and lipid metabolism in disrupting ER homeostasis

While PTT alone can induce ERS through thermal and oxidative damage, tumor cells often promote survival by activating adaptive UPR pathways (148). To overcome this resilience, synergistic strategies combining PTT with ER-targeted agents have emerged to amplify proteotoxic stress, disrupt lipid metabolism, and trigger ICD.

#### 4.2.1 Inducing ER stress and UPR

PTT-induced hyperthermia and ROS compromise ER membrane integrity and chaperone function, resulting in the accumulation of misfolded proteins. However, the adaptive UPR often attenuates this stress, maintaining cell survival (55). To convert ERS from a prosurvival into a prodeath signal, PTT is combined with UPR pathway inhibitors. The UPR contains multiple signaling branches, among which the PERK-CHOP pathway is the principal proapoptotic pathway. For instance, codelivery of PERK or IRE1α inhibitors with PTAs prevents the recovery of ER homeostasis, driving cells toward apoptosis through the upregulation of CHOP and the downregulation of Bcl-2 (149, 150). Additionally, agents that deplete ER calcium stores can exacerbate calcium dyshomeostasis, leading to mitochondrial calcium overload and enhancing the cytotoxicity of PTT (151).

#### 4.2.2 Disrupting ER-mediated lipid biosynthesis

Beyond protein homeostasis, the ER is the primary site for *de novo* lipid synthesis, including fatty acids, phospholipids, and cholesterol, which are essential for membrane integrity, signaling, and energy storage in rapidly proliferating tumor cells (152). Key enzymes such as FASN, ATP-citrate lyase (ACLY), and stearoyl-CoA desaturase 1 (SCD1) are frequently upregulated in cancers and contribute to metabolic reprogramming. The inhibition of enzymes such as FASN may disrupt the lipid balance of the ER and trigger lipotoxic stress (153). However, no relevant literature has explored or confirmed that PTT inhibits the enzymatic activities of FASN, ACLY, or SCD1. Therefore, nanoplatforms combining PTAs with lipid metabolism inhibitors such as C75 or SB-204990 have been developed to enable the targeted disruption of lipid biosynthesis. The thermal effect from PTT further exacerbates lipid bilayer disruption and impairs membrane fluidity (154). For example, graphene-based nanosystems loaded with SB-204990 have been shown to synergize with PTT to induce lipid starvation, impair membrane fluidity, and suppress tumor growth in oral squamous cell carcinoma (154). This dual strategy not only deprives tumors of lipids required for proliferation but also potentiates thermal damage by compromising membrane integrity and repair mechanisms. Furthermore, PTT can also target lipid structures through oxidative stress. ROS generated during PTT can attack unsaturated fatty acids in the cell membrane, triggering lipid peroxidation (53). However, polyunsaturated fatty acid (PUFA)-containing lipids fail to trigger lethal lipid peroxidation upon attack by ROS (155). Therefore, enhancing the abundance of PUFA-containing lipids in membranes can significantly increase the sensitivity to ferroptosis. Evidence suggests that SLC47A1 inhibits ferroptosis by downregulating the esterification of PUFAs into cholesterol esters (156, 157). Thus, enriching PUFA-containing lipids by inhibiting SLC47A1 expression can strengthen lipid metabolic processes to promote ferroptosis. The latest research has found that lipid peroxidation end products such as 4-hydroxynonenal are cytotoxic and can further damage organelles such as the ER, exacerbating the metabolic crisis (24). Hepatoma cells upregulate aldehyde-metabolizing enzymes to detoxify cytotoxic aldehydes such as 4-hydroxynonenal (4-HNE) (158, 159). Furthermore, in hepatoma cells, 4-HNE is rapidly conjugated to GSH and exported extracellularly, thereby reducing oxidative damage (159). These adaptive responses provide new targets for PTT to inhibit lipid metabolism in tumors. In contrast, sustained iron-mediated lipid peroxidation leads to ferroptosis. A study demonstrated that PTT combined with chemodynamic therapy (CDT) can lead to the accumulation of lipid peroxides, accompanied by the depletion of GSH and the inactivation of glutathione peroxidase 4 (GPX4), a key feature of ferroptosis (24). During this process, the thermal effect produced by PTT enhances the catalytic efficiency of CDT. At the same time, the thermal effect disrupts the redox balance of cells by consuming GSH, thereby eliminating the inhibitory effect of GPX4 on lipid peroxidation. However, most existing studies remain at the stage of observing lipid peroxidation without elucidating its underlying molecular mechanisms or downstream metabolic consequences (53).

### 4.3 Cytosolic metabolic enzymes and transporters as therapeutic targets

The cytoplasm is the primary site for cellular processes, including signal transduction, protein translation, and stress response. Targeting metabolic reprogramming in the cytoplasm of tumor cells with PTT has become an emerging strategy for enhancing therapeutic efficacy. These strategies disrupt the metabolic homeostasis of tumor cells by targeting metabolites, enzymes, signaling pathways, or organelle functions within the cytoplasm, thereby overcoming thermotolerance and achieving synergistic effects.

#### 4.3.1 Targeting metabolic enzymes in the cytosol

**Figure 5:**
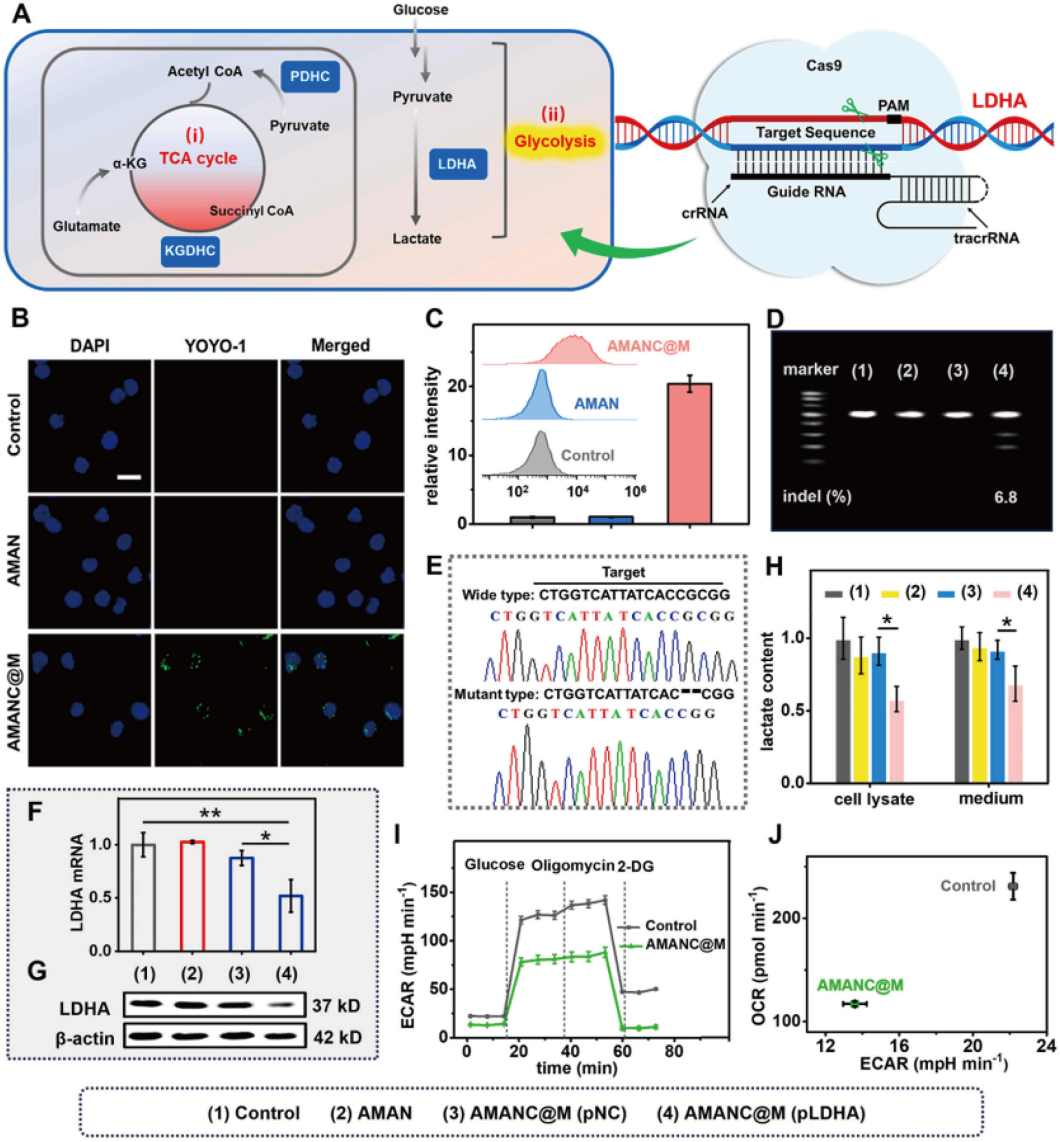
AMANC@M precisely induced LDHA genome editing and then glycolysis inhibition as an “energy nanolock”. **(A)** Schematic of simultaneous inhibition of the TCA cycle (i) and glycolysis (ii). Inhibition of glycolysis was achieved by knocking down LDHA using CRISPR/Cas9 technology. **(B)** CLSM images (nuclei: blue; scale bar: 20 μm); **(C)** flow cytometry analysis of 4T1 cells after different treatments. CRISPR/Cas9 plasmid was labeled with YOYO-1 (green). **(D)** T7E1 assay on the genomic DNA extracted from 4T1 cells after different treatments. **(E)** The DNA sequencing results of LDHA were detected in mutant colonies treated with AMANC@M (pLDHA) compared to untreated cells. **(F)** qPCR assay on LDHA mRNA in 4T1 cells after different treatments. **(G)** Western blot analysis of LDHA expression in 4T1 cells after different treatments for 72 h. **(H)** Normalized lactate concentrations in cell lysate and culture medium after different treatments for 48 h. **(I)** ECAR levels; **(J)** metabolic phenotypes of 4T1 cells. The groups were as follows (1): control (2), AMAN (3), AMANC@M (pNC), and (4) AMANC@M (pLDHA). All data are expressed as mean ± SD (*n* = 3). ^**^p < 0.01; ^*^p < 0.05 (167). Copyright 2024, Elsevier.

The cytosol is the main site of various metabolic pathways such as glycolysis and the PPP. Synergistic inhibition of rate-limiting enzymes in these pathways can induce a dual energy crisis and oxidative stress, thereby overcoming thermotolerance to PTT. The inhibition of glycolytic enzymes significantly suppresses ATP production and downstream HSP expression. For instance, inhibitors that target HK2, such as 3-bromopyruvic acid, inhibit the phosphorylation of glucose in the cytosol (160, 161). Beyond small molecules, molecularly imprinted polymers (MIPs) with specific recognition for target proteins provide innovative strategies for inhibiting active sites, including those of HK2 (162). Similarly, PKM2 in the cytosol is also an important target for sensitizing tumors to PTT by modulating metabolic reprogramming. Silencing PKM2 can disrupt the glycolytic pathway of tumor cells, diminish their energy metabolism, and reduce thermotolerance to PTT (67). For instance, downregulating the glycolytic enzyme PKM2 has been shown to not only inhibit glycolysis but also reduce HSP expression, thereby overcoming thermotolerance and improving PTT efficacy (163). LDHA in the cytoplasm is another enzyme in the glycolytic pathway (164–166). Emerging strategies further demonstrate that precise downregulation of LDHA via gene-editing tools such as CRISPR/Cas9 can significantly amplify the efficacy of PTT. For instance, in TNBC, nanoparticle-mediated CRISPR/Cas9 delivery for LDHA knockout has been demonstrated to inhibit glycolysis in synergy with PTT to promote tumor cell apoptosis (167) (**Figure 5**). However, PTT-induced inflammation may promote tumor metastasis, while lactate produced by tumor cells can foster an immunosuppressive TME, collectively contributing to therapeutic resistance. Recent studies have shown that PTT can alleviate therapy-induced inflammatory cascades, such as epithelial-mesenchymal transition, by neutralizing cytokines and eliminating the tumor-derived secretory factors as “nutrients” supplied to polymorphonuclear leukocytes (20). However, focusing solely on the cytokines tumor necrosis factor alpha (TNF-α) and interleukin (IL)-6 offers a limited perspective. The therapeutic synergy may also involve other pathways, such as those activated by interferon gamma (IFN-γ) signaling through its receptor on M1 macrophages.

To reduce the antioxidant capacity of tumors, cotargeting PPP enzymes with PTT has emerged as a promising strategy. G6PD, the rate-limiting enzyme of the PPP, has garnered significant attention as a therapeutic target for synergistically enhancing PTT efficacy. The combination of PTT with G6PD inhibition disrupts NADPH homeostasis, involving both nanozymes that rapidly consume NADPH and generate ROS via photothermally enhanced NOX-like activity and pharmacological G6PD inhibition that blocks NADPH regeneration. This coordinated disruption exacerbates oxidative stress and potentially triggers ferroptosis (168, 169). For instance, the bioorthogonal nanozyme designed by Huang has been proven to downregulate the levels of G6PD and DPP, increase tumor oxidative stress, and enhance the efficacy of mPTT (169). In addition to the NADPH regeneration system, highly expressed glutathione *S*-transferases (GSTs) in the cytoplasm can mitigate PTT-induced oxidative damage. These enzymes catalyze the conjugation of GSH with lipid peroxidation products, such as acrolein, facilitating their elimination (170). Szukalska et al. found that the level of GST activity in kidneys treated with nanomaterials upon laser irradiation decreased significantly by approximately 58% (45). Therefore, GST gene silencing or pharmacological inhibition mediated by nanocarriers can establish a synergistic loop with PTT, potentiating therapeutic efficacy. Furthermore, the JNK signal pathway enhanced by GST inhibition can intersect with the PTT-induced apoptotic pathway, such as the death receptor DR5/caspase-3 pathway, further promoting apoptosis (170). PTT and GSTs may synergistically regulate metabolic changes related to inflammation, such as the shift from proinflammatory metabolism to anti-inflammatory or proapoptotic metabolism. Importantly, the effect of PTT on GST activity is closely related to the target organ and the specific composition of the nanomaterials. For example, the level of GST activity in the brains of mice that received PDA@DG3@PEG@FA@doxorubicin (DOX) nanoparticles along with phototherapy was 72% higher than that of the group that received only PDA@DG3@PEG@FA nanoparticles (171). Another study found that the inactivation of glutamylcysteine ligase inhibits the synthesis of endogenous GSH, promoting ferroptosis in cancer (172). This also indicates that glutamylcysteine ligase may be a new target for the synergistic regulation of tumor metabolic reprogramming by PTT. Furthermore, methylglyoxal (MGO), as a byproduct of glycolysis, can modify proteins and damage DNA, affecting cellular function. However, tumor cells rely on glyoxalase 1 (GLO1) and glyoxalase 2 (GLO2) to detoxify MGO for survival (173). Studies have shown that inhibiting GLO1 can lead to the accumulation of MGO within tumors, revealing the potential of MGO as a therapeutic target for tumors (174).

#### 4.3.2 Targeting nutrient transporters of the cytoplasmic membrane

The cytoplasmic membrane is the primary interface for material exchange between the cell and its extracellular environment. Targeting nutrient transporters on the membrane represents an upstream strategy to disrupt the intracellular substrate supply, thereby synergizing with PTT by inducing metabolic stress. Small-molecule inhibitors such as diclofenac can bind to specific amino acid residues within the GLUT1 channel, such as GLU380 and ASN288, effectively blocking glucose uptake (175). This strategy deprives tumors of glycolytic substrates at their source, leading to a cascade of ATP depletion and subsequent downregulation of HSP expression. Lactate transporter monocarboxylate transporter 1 (MCT1) and alanine–serine–cysteine transporter 2 (ASCT2) are also targets for the synergistic regulation of glutamine metabolism with PTT (176). Inhibition of MCT1 leads to the accumulation of lactate in the cytoplasm, acidifying the intracellular environment. Concurrently, it reduces oxygen consumption required for adaptation to hypoxia, thereby indirectly improving the TME and enhancing the efficacy of other therapies (19). For instance, You et al. conducted a study on the combined application of cervical cancer biomarker SLC16A1/3 and PTT, demonstrating a novel synergistic approach for cervical cancer (177). However, literature on combining ASCT2 inhibition with PTT to modulate tumor metabolism remains scarce. This gap may be explained by the fact that hyperthermic stress could reduce protein translation by inhibiting the mTORC1 signaling pathway while upregulating the expression of solute carrier family 3 member 2 (SLC3A2) (178). Thus, it is a promising direction to investigate whether PTT upregulates SLC3A2 and its partner transporters such as LAT1 and xCT. This line of inquiry could reveal whether inhibiting these compensatory pathways synergizes with ASCT2 blockade to more effectively disrupt tumor metabolism.

### 4.4 Autophagy inhibition and metabolic stress in lysosomal disruption

Lysosomes promote tumor metabolic reprogramming through autophagy and nutrient sensing, contributing to biosynthesis and energy homeostasis. Disrupting lysosomal activity can trigger metabolic crisis in tumor cells. Lysosomes are the final stage of the autophagy process. PTT induces protective autophagy and promotes tumor cell survival, as evidenced by increased levels of LC3-II, CFP-LC3 dots, and expression of ATG3 and Beclin-1 (179, 180). The synergistic strategy blocks this metabolic adaptation pathway by delivering autophagy inhibitors such as chloroquine or by disrupting autophagosome-lysosomal fusion. A key strategy involves disrupting the acidic environment of lysosomes, thereby inhibiting the fusion of autophagosomes with lysosomes and the degradation of their contents. Studies have found that weakly basic drugs such as chloroquine and hydroxychloroquine are protonated and enriched in the cavity of lysosomes, neutralizing the H^+^ in the cavity, leading to an increase in pH and inactivation of cathepsin B (181, 182). This strategy disrupts the self-protection mechanisms of cancer cells under hyperthermia and ROS, amplifying the synergistic effect of PTT and CDT. Consistently, combining PTT with the autophagy inhibitor primaquine (PQ) was shown to preferentially block autophagosome-lysosome fusion by altering lysosomal pH (180). Beyond pharmacological intervention, the fusion process is regulated by the endosomal sorting complexes required for transport (ESCRT). The inhibition of autophagosome-lysosome fusion mediated by ESCRT is crucial for cell survival. For instance, Wu et al. demonstrated that ablation of ESCRT-0-STAM2 inhibits autophagosome-lysosome fusion and protects skin cells from heat-induced death through interaction with HSPA1A (183). These findings underscore the importance of HSPA1A-ESCRT-0/STAM2-mediated autophagy in thermoresistance, highlighting a clinically relevant mechanism for PTT.

Beyond small molecules, proteins maintaining lysosomal acidity present alternative targets. Research indicates that the chloride channel-3 (ClC-3) protein is a critical Cl^−^/H^+^ exchanger for maintaining the acidic environment of lysosomes (179). Inhibiting the function or expression of ClC-3 represents another target for lysosomal alkalization. For example, Meng et al. demonstrated that ClC-3 silencing inhibited protective autophagy, thereby sensitizing tumor cells to chemophotothermal therapy and reducing DOX resistance by impairing lysosomal acidification (179). Similarly, vacuolar-type H^+^-ATPase (V-ATPase) serves as a primary proton pump for the acidic environment of lysosomes. Its inhibition prevents tumor cells from triggering metabolic reprogramming through the AMPK pathway, rendering them more susceptible to cell death (184). Therefore, combining specific V-ATPase inhibitors with PTT may enhance lysosome-dependent cell death. Furthermore, inhibiting the autophagy-lysosomal pathway can lead to misfolded or damaged proteins relying more on UPS for degradation (107). Therefore, this interplay requires consideration of the UPS when PTT is combined with autophagy-lysosomal pathway inhibitors.

In addition to pharmacological inhibitors, nanomaterials can be engineered to disrupt the lysosomal microenvironment. During endocytosis, V-ATPase translocates protons from the cytoplasm to the endosome, thereby acidifying the endosome (185). Therefore, some high-molecular weight nanocatalysts such as polyethyleneimine utilize the proton sponge effect to capture H^+^ ions through a large number of protonable groups, resulting in lysosomal osmotic swelling and rupture (107). Other studies indicate that self-assembled nanofibers targeting carbonic anhydrase IX (CA IX) expand in size as the pH decreases after entering cells through endocytosis. This effect subsequently causes intracellular acid vesicle damage and the inhibition of protective autophagy (186). Photochemical internalization (PCI) represents another powerful strategy for spatiotemporally controlled intracellular release, which depends on the light activation of a photosensitizer localized on endo- or lysosomal membranes, generating ROS that disrupt vesicular integrity (187). This principle can be effectively combined with photothermal materials. For instance, near-infrared irradiation of reduced graphene oxide-PEI nanocomposites generates localized heat, which facilitates their endosomal escape and thereby achieves highly efficient gene transfection (185). However, there are few studies on the regulation of metabolic reprogramming by PTT in coordination with lysosomal dysfunction inducers.

### 4.5 Targeting the HSF1-HIF-1α synergy to block metabolic adaptation

To cope with hyperthermia and ROS induced by PTT, the cell nucleus coordinates adaptive metabolic reprogramming through a series of stress-responsive transcription factors. The ROS produced by PTT inhibit the activity of prolyl hydroxylase (PHD), leading to the stabilization of hypoxia-inducible factor-1α (HIF-1α) and its nuclear translocation, thereby activating it under normoxia (188). Meanwhile, localized hyperthermia triggers HSF1 activation, causing it to dissociate from the inhibitory complex and be transported into the nucleus. The interaction between activated HSF1 and HIF-1α promotes thermotolerance. Nuclear HIF-1α upregulates the expression of glucose transporter GLUT1 and glycolytic enzymes such as HK2, PKM2, LDHA, and PDK1, thereby enhancing glycolytic flux (63). For instance, inhibiting HIF-1α suppresses proliferation and promotes apoptosis in hepatocellular carcinoma by disrupting the glycolysis mediated by PKM2 (189). On the other hand, activated HSF1 initiates the transcription of HSPs, which repair damaged proteins and inhibit apoptosis, thereby enhancing the thermotolerance of tumor cells (190). The mutual regulation between HIF-1α and HSF1 forms an interdependent network, where HIF-1α can directly or indirectly promote the expression of HSPs (191). Conversely, HSF1 enhances the mRNA stability of metabolic genes through transcription factors such as Hnrnpa2b1 and participates in pathways such as FAM3A-PANX1-ATP to affect energy metabolism and biosynthesis (192, 193). Therefore, targeting a single factor often yields limited efficacy.

Targeting HIF-1α or its upstream regulators with small interfering RNA (siRNA) or small molecules represents a primary strategy to disrupt adaptive tolerance. However, such strategies mainly interfere with hypoxia adaptation and often fail to address the metabolic synergy with PTT. Another approach is to target the transcriptional coactivators of HIF-1α, such as SRC-3, to synergize with PTT. For instance, bufalin is combined with mPTT to target SRC-3/HIF-1α, thereby suppressing GLUT1 expression and glycolytic flux. This pharmacological blockade of hypoxia adaptation synergizes with PTT to enhance thermosensitivity (63). However, merely inhibiting the HIF-1α axis is insufficient to completely reverse the thermotolerance and metabolic remodeling maintained by the HSF1 axis. For the HSF1 axis, targeting the downstream proteins of the HSPs has become an important direction for enhancing the efficacy of PTT. One of the methods is to use small-molecule HSP inhibitors, which compete for the ATP binding sites in the N- or C-terminal domains to deprive the protein of its ATP binding ability (194). For example, gambogic acid is an HSP inhibitor that is combined with PTT to block the chaperone function. This pharmacological inhibition of ATP-dependent HSPs depletes the chaperone machinery that tumor cells rely on to survive thermal injury, an effect not induced by PTT alone (195). Functional redundancy within the HSP family also limits single-target approaches, providing a rationale for combination therapies, such as HSP90/HSP70 (196). The CRISPR screening technology has been utilized to achieve localized therapeutic gene expression, including in gene-directed enzyme prodrug therapy (GDEPT) and CRISPR-Cas9 systems activated during PTT (197). The mild photothermal effect produced by PBDTQ could prompt the release and activation of a suicide gene plasmid (pSG), thereby activating the HSP70 promoter and subsequently upregulating the expression of the suicide gene triggered by the HSP70 promoter. Notably, tumor hypoxia synergistically amplifies HSF1-driven promoter activity by stabilizing HIF-1α, which co-occupies heat shock elements and further enhances tumor selectivity (198). Furthermore, inhibiting glycolysis can significantly reduce ATP levels and downregulate the expression of HSPs, thereby enhancing the efficacy of PTT (199). These strategies aim to suppress tumor thermotolerance while simultaneously interfering with metabolic reprogramming by combining with PTT. In conclusion, dual targeting of the HSF1 and HIF-1α pathways represents a promising strategy to synergistically disrupt tumor thermotolerance and metabolic adaptation, thereby overcoming PTT resistance.

### 4.6 PTT targeting multiorganelle to regulate tumor metabolism

The adaptability of organelles and their interactions often limit the efficacy of single-target strategies. For instance, the enzymatic environment of lysosomes and various hydrolases can degrade the drugs, leading to treatment failure (200). The P-glycoprotein (P-gp) on the cell membrane can expel chemotherapy drugs from the cell through ATP produced by mitochondria (201). To overcome these interconnected defense mechanisms, simultaneously disrupting the functions of multiorganelle, such as lysosomes, mitochondria, ER, and Golgi apparatus (GA), has become a new approach to interfering with tumor metabolism. For example, Liu et al. constructed a folate (FA)-targeted bovine serum albumin (BSA) nanoparticle, which simultaneously carried a copper-5-nitro-8-hydroxyquinoline complex (Cu/NQ), IR780, and DOX. The FA-BSA/Cu/NQ/IR780/DOX (FA-BCNID) nanoparticle was designed to target multiorganelle individually. After endocytosis occurred, the Cu/NQ complex destroyed the lysosomal membrane, allowing the nanoparticles to escape into the cytoplasm and preventing drug degradation. IR780 generated ROS and hyperthermia through PTT, causing mitochondrial depolarization and ATP depletion, thereby inhibiting the ATP-dependent P-gp efflux pump. This multiorganelle cascade reaction, including lysosomal escape, mitochondrial dysfunction, and nuclear DNA damage, was significantly enhanced *in vitro* and *in vivo* compared to single-target strategies (202).

Other studies further expand the scope of multiorganelle targeting strategies, including the ER and GA. Mohan et al. demonstrated that PTT mediated by gold nanoparticles conjugated with albumin (Alb-GNPs) could specifically target the GA and ER in hepatoma cells. After internalization, Alb-GNPs triggered the collapse of the GA structure upon PTT treatment, followed by severe expansion of the ER membrane and subsequent activation of the caspase-3 apoptotic pathway (203). This work provides early evidence that the heat induced by PTT can directly damage the Golgi-ER network. In the context of glioblastoma (GBM), a ruthenium-based nanoplatform (Fe_3_O_4_@PDA@Ru) was localized within multiorganelles after internalization, including ER, GA, lysosomes, and mitochondria (24). Under laser irradiation, this platform generated ¹O_2_ and heat, leading to a comprehensive stress response, including glutathione depletion, GPX4 inactivation, ferroptosis, and the upregulation of the ERS sensor ATF6. This multi-target combined strategy against organelles, combined with PTT, ferroptosis, and ERS, is more effective in killing GBM cells than single-target treatments. In summary, these studies emphasize the therapeutic potential of simultaneously targeting multiorganelles. Unlike strategies that target only a single organelle, a multiorganelle targeting strategy can disrupt the compensatory interactions between organelles and overcome multiple resistance mechanisms simultaneously. **Table 1** summarizes representative examples of PTT-based combination strategies categorized by their primary organelle targets. Notably, a substantial number of studies have focused on mitochondria- or cytosol-targeted photothermal nanoplatforms.

**Table 1:** Representative examples of organelle-targeted PTT combination strategies.

Primary organelle	Nanoparticle	Treatment modalities	Tumor models	Immuno outcomes	Translational limitations	Ref
Mitochondria	TM@CD326hOMV	Copper depletion strategy/PTT/PD-1L antibody	4 T1	CD8+ T↑/NK infiltration	Diet-Cu abolishes efficacy; EpCAM heterogeneity	(204)
Mitochondria	P1+P2@COF@F127-D	PTT/cuproptosis	B16	ICD↑; DC maturation; CD8+ T infiltration↑; Treg↓	Cu chronic toxicity; SLC7A11 dependency; MOF degradability	(141)
Mitochondria	AuNRs@Cu2O	Cuprotosis/PTT	4T1/B16F10	ICD↑; DC maturation; CD8+ T↑; M1/M2↑; TCM↑; cytokines↑	Cu neurotoxicity; AuNRs nondegradable; NIR-II depth	(205)
Mitochondria	PAI@CB839	PTT/glutaminase inhibitor/ferroptosis	4 T1	ICD↑; DC maturation; CD8+ T↑; Treg↓; M1/M2↑	NIR-I depth; hypoxia heterogeneity; photobleaching	(142)
Mitochondria	DECaNG	Chemotherapy/PTT/imbalance of mitochondrial Ca2+ homeostasis	MCF-7	ICD↑; DC maturation; CD8+ T↑; M1/M2↑; TCM↑	Cu neurotoxicity; AuNRs nondegradable; NIR-II depth	(205)
Mitochondria	ZIF-8@ArBu&ICG@MPN@HA	CDT/PTT	HepG2	CD4+ T↑	Lack long-term toxicity and biodistribution data	(206)
Mitochondria	M@CD/MTO	Ferroptosis/PANoptosis/PTT	4 T1	ICD↑; DC maturation; CD4+ T↑; M1/M2 repolarization; IFN-γ/TNF-α/IL-6↑; Treg↓	Ce long-term toxicity unknown; MTO cardiotoxicity risk	(207)
Mitochondria	CuS@axitinib-SiO2@2-deoxy-d-glucose(2-DG)CaCO3-RGD	PTT/CDT/starvation therapy	4 T1	M2→M1 polarization; CD8+ T↑; IL-6↑; IL-10↓	Cu2+/Ca2+ toxicity; lack an active immune checkpoint evaluation	(208)
Cytosol	CD@Pt SA/NC@DOX	Nanozyme catalytic therapy/low-temperature PTT/CT	4T1	TNF-α↑	NIR-I depth; unclear biodegradability	(209)
Cytosol	IB@Fe-ZIF8@PDFA	Ferroptosis/PTT/CDT	4TI	TNF-α↑	NIR-I depth; mPTT precision; H2O2/hypoxia limits; Fe/Zn toxicity	(210)
Cytosol	GNR/HA-DC	Starvation therapy/PTT	HeLa/MCF-7	TNF-α, IL-6, IL-1↓ in LPS-stimulated RAW 264.7	CTAB risk; multistep	(211)
Mitochondria/cytosol	HBI NDs	PTT/immune checkpoint blocker	4 T1	CTL infiltration↑; TNF-α/IFN-γ↑; PD-L1/IDO-1↓; lung metastasis↓	Complex self-assembly; lack long-term toxicity data	(212)
Mitochondria/cytosol	APHA@CM	SDT/PTT/CAR T-cell therapy	NALM 6	ICD↑; DC maturation↑; macrophage repolarization; CD4+/CD8+ proliferation; Treg↓; T_EM/T_CM↑	US + NIR-II limit clinical translation; Ag+/Au long-term toxicity	(162)
Mitochondria/cytosol	PsiL@M1M	PTT/anti-inflammatory	4 T1	ICD↑; DC maturation↑; CD8+↑; Treg/MDSC/TAM↓; Tcm/Tem↑	Complex assembly; dual dosing regimen required; potential immunogenicity of M1M; long-term safety unclear	(20)

## 5 PTT-induced metabolic disruption alters the physicochemical landscape of the TME

Both hypoxia and redox imbalance in the TME contribute to tumor development and serve as therapeutic targets (213). Studies have shown that hypoxia inhibits mitochondrial peptide deformylase (HsPDF) by upregulating tRF-3Thr-CGT, resulting in the inhibition of protein translation encoded by mtDNA, thereby suppressing OXPHOS. Hypoxia also disrupts the expression of OXPHOS proteins encoded by nuclear DNA and mtDNA (214). Moreover, oxidative stress in the TME directly damages mitochondria, disrupting their dynamics, redox balance, and mtDNA signal transduction (215). However, there are few studies on the changes in tumor hypoxia levels caused by PTT-induced mitochondrial dysfunction. Accordingly, the synergistic strategies with PTT reprogram the metabolism of organelles in tumor cells by altering the signals in the TME, such as oxygen tension and pH.

At the molecular level, the intersection of hypoxia and ROS signaling occurs within the nucleus. ROS stabilize HIF-1α by inhibiting PHD activity (216). Studies have shown that H_2_O_2_ oxidizes the cysteine residue (Cys326) on the PHD2 protein, leading to dimerization and loss of hydroxylase activity. Inactivated PHD2 cannot label HIF-1α for ubiquitination and degradation, leading to HIF-1α accumulation in the cell nucleus (217). HIF-1α in turn regulates genes involved in metabolism and ROS generation, thereby contributing to a positive feedback loop (218). Photothermal synergy strategies are designed to disrupt this feedback loop. For instance, the oxygen production strategy based on nanocatalysts aims to do more than simply increase local oxygen concentration (162). This strategy actively destabilizes HIF-1α, whose stability is maintained by the hypoxia-ROS crosstalk interaction. This measure can indirectly alleviate the transcriptional inhibition of genes related to mitochondrial biosynthesis and OXPHOS, thereby sensitizing tumor cells to PTT-induced metabolic stress.

Mitochondria are not only the core effectors of hypoxia-ROS interactions but also the key links in their cascade amplification. Hypoxia causes dysfunction of the ETC and leakage of ROS (218). These leaked ROS further damage the mitochondria, contributing to a vicious cycle (219). Research has found that exogenous ROS induces the overexpression of dynamin-related protein 1, disrupting mitochondrial homeostasis (139). Combining CDT with PTT intensifies the vicious cycle by introducing exogenous catalysts such as iron-based nanozymes (220). These catalysts convert the high concentrations of H_2_O_2_ accumulated in the TME due to metabolic abnormalities into more toxic hydroxyl radicals. However, the increase in ROS upregulates the expression of HSPs and weakens the photothermal efficacy (221). Meanwhile, nanotherapy aims to deplete GSH to weaken the defense mechanisms of tumor cells against such oxidative stress through TME-responsive strategies. The most common strategy is to utilize nanomaterials to mimic the activity of enzymes such as glutathione oxidase (GSH-OXD), converting reduced GSH into its oxidized form (GSSG), thereby consuming GSH (222). Some metal ions consume GSH through redox reactions or complexation with it (223). Another strategy is to design nano-prodrugs that can be activated by γ-glutamyl transpeptidase (GGT) overexpressed in the TME or acidic pH, specifically releasing thiol-reactive molecules, consuming GSH on the cell surface, or interfering with its uptake (224). Both strategies involve oxidative stress in the TME exceeding a critical threshold, thereby triggering mitochondrial lipid peroxidation and ferroptosis.

In the ER, improving hypoxia and oxidative stress can alleviate ERS, thereby inhibiting the excessive activation of UPR branches such as IRE1-XBP1 and PERK. The reduction of ERS also inhibits the release of brain-derived neurotrophic factor precursors (proBDNF) by cancer cells, thereby suppressing cancer progression (225–227). Therefore, a potential strategy to enhance PTT is to inhibit the ERS pathway while simultaneously increasing local oxygen concentration and ROS levels within the tumor. Notably, tumor cells respond to hypoxia and redox reactions by secreting vesicles, such as exosomes, for complex intercellular information exchange. For example, gastric cancer cells under hypoxia secrete exosomes enriched in miR-301a-3p, which suppresses PHD3 expression in recipient cells, leading to sustained HIF-1α stabilization even in better-oxygenated regions (228). Thus, future PTT-combination therapies should simultaneously target oxygen tension and intercept such organelle-derived, exosome-mediated adaptive responses to achieve comprehensive hypoxia normalization.

The acidification of the TME promotes the growth of cancer cells, providing a target for the coordinated regulation of tumor metabolism by PTT. Under hypoxia, the activation of HIF transcription factors promotes the Warburg effect, shifting tumor cell metabolism toward glycolysis, which leads to the accumulation of lactate and acidification of the microenvironment (229, 230). To support high metabolic demands, lysosomal activity is significantly upregulated, and the V-ATPase pump continuously exports protons into the lysosomal lumen or extracellular space (231). Together, these processes contribute to the formation of an acidic microenvironment through plasma membrane transporters (232). This acidity not only facilitates the degradation of the extracellular matrix (ECM) and tumor metastasis but also suppresses CTLs and NK cells while promoting angiogenesis through VEGF (233, 234). The most common strategy is to neutralize the acidity of the tumor, relieve the suppression of the immune system, and reverse the tumor-promoting effects, thereby enhancing the efficacy of PTT. For instance, manganese dioxide (MnO_2_) nanoparticles can react with H^+^ in the TME to increase the pH value and enhance PTT efficacy (235). Critically, the increase in the external pH value can alleviate the burden of the lysosomal V-ATPase pump operating at continuous overload to separate protons. Notably, CA IX, overexpressed in hypoxic tumors, facilitates the transmembrane efflux of acidic metabolites, thereby protecting cancer cells from intracellular acidosis during hypoxia-induced metabolism (186). Therefore, inhibiting CA IX with carbonic anhydrase inhibitors (CAIs) represents a promising strategy to disrupt pH homeostasis in tumors. For example, CAI-modified pyrite nanoparticles (FeS_2_-PEG-CAI NPs) disrupt lysosomal pH regulation by increasing the intracellular proton load, which can synergize with V-ATPase inhibition to enhance the efficacy of PTT (236). However, research remains scarce on how CAI-induced intracellular acidification affects the functions of organelles such as mitochondria.

## 6 The tumor immune microenvironment reshaped by PTT-induced organelle stress

**Figure 6:**
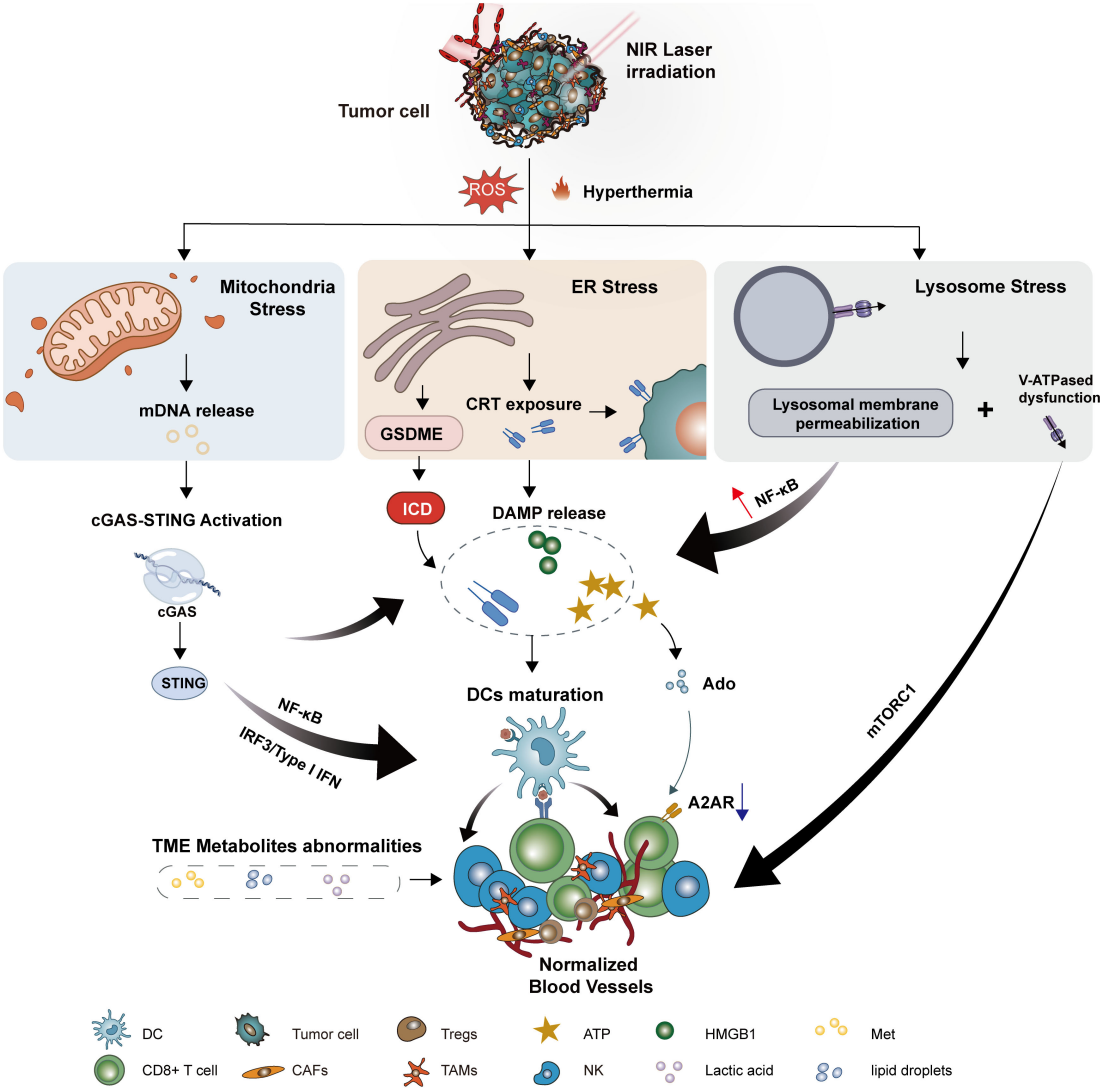
PTT-induced organelle stress reprograms the tumor immune microenvironment. This schematic diagram illustrates that the hyperthermia and ROS triggered by PTT simultaneously act on multiple organelles, such as the mitochondria, the ER, and the lysosomes, to modulate antitumor immunity.

The organelle stress and metabolic disruption induced by PTT extend beyond the tumor cell to reshape the TME (**Figure 6**). The temperature of PTT determines the cell death modality. In traditional PTT, when the temperature exceeds 50°C, tumor cells undergo swelling, cell membrane rupture, and disintegration of organelles (28). The disruption of cell membrane function will lead to the release of excessive inflammatory mediators, such as IL-6, HSPs, and nucleic acids, enhancing the infiltration of immunosuppressive cells and inhibiting T-cell responses. At the same time, excessive heat will also damage the structural integrity of TAAs and DAMPs, making traditional PTT less effective in immunotherapy (14). However, under mPTT at a lower temperature, relatively intact organelles, especially mitochondria and ER, can release immunogenic signals, including mtDNA and DAMPs. These signals can activate the cGAS-STING pathway and induce ICD, thereby initiating innate and adaptive immune responses. In addition, TME-derived metabolites, such as glutamine, lipids, methionine, and lactate, reprogram the metabolism of infiltrating immune cells, altering mitochondrial function, lipid accumulation, and mTORC1 signaling, thereby modulating their effector functions. We discuss how PTT-induced immunogenic signals are counteracted by immune checkpoints and how combinatorial strategies targeting these interconnected pathways can maximize therapeutic efficacy.

### 6.1 Innate immune signaling and immunogenic cell death triggered by organelle stress

**Figure 7:**
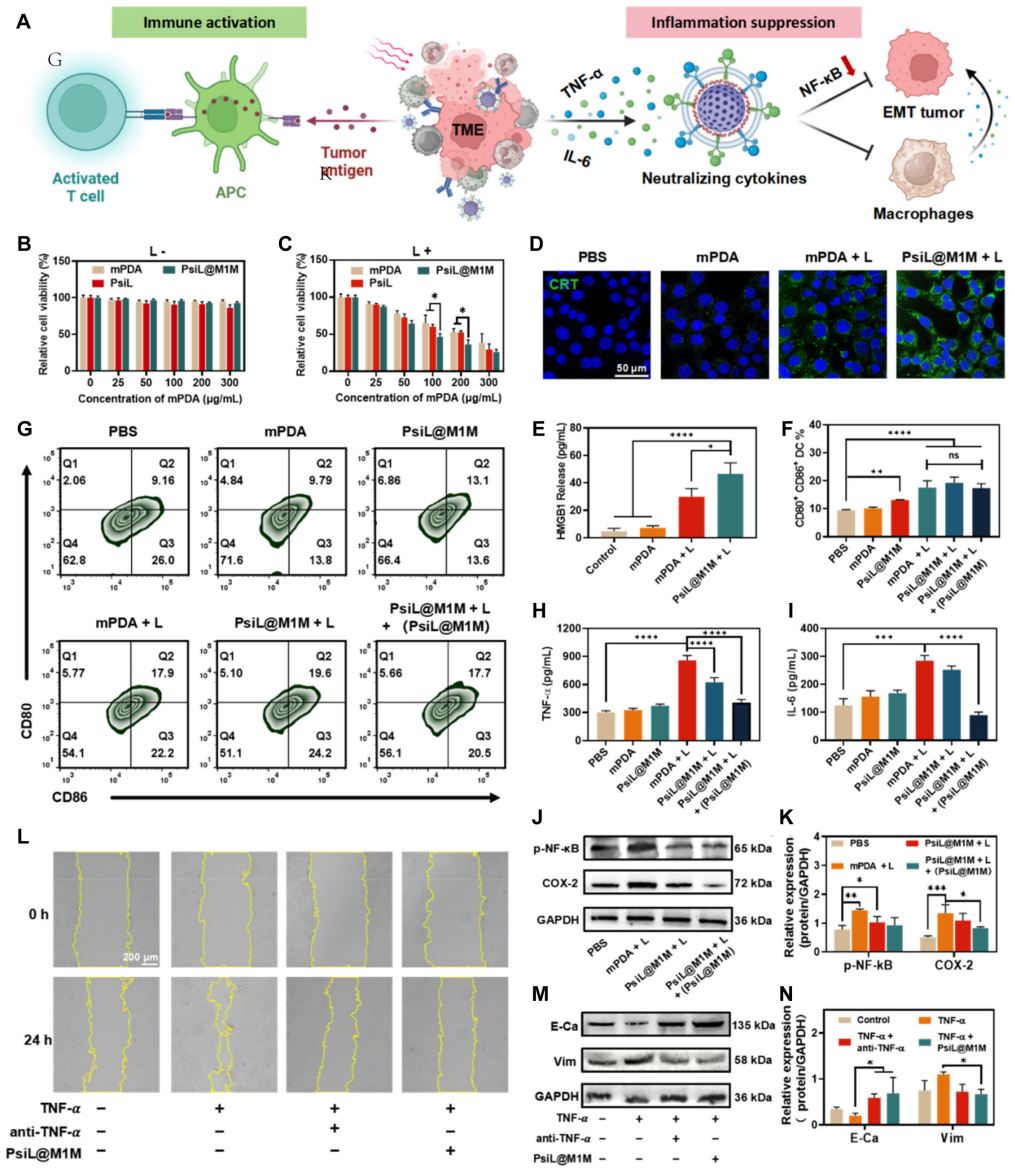
Hypoinflammatory photoimmunotherapy mediated by PsiL@M1M *in vitro*. **(A)** Schematic representation of PsiL@M1M-mediated immune activation and inflammation suppression. Viability of 4T1 cancer cells treated with PsiL@M1M and relevant controls **(B)** in the dark and **(C)** with laser irradiation. **(D)** Surface-exposed CRT in 4T1 cells and **(E)** HMGB1 release after treatment under different conditions. **(F)** Percentages of CD80- and CD86-positive DCs under different stimulation conditions and **(G)** representative flow cytometry plots. Concentrations of **(H)** TNF-α and **(I)** IL-6 in the supernatants of DC2.4 cells cocultured with 4T1 cells treated with different nanoformulations. **(J)** Western blot analysis and **(K)** corresponding quantification of inflammation-associated proteins. **(L)** Cell migration was evaluated using the scratch wound healing assay. **(M)** Western blot analysis and **(N)** corresponding quantification of EMT-associated proteins (20). Copyright 2025, Elsevier.

The oxidative burst induced by PTT damages mitochondria and triggers the release of mtDNA, enhancing the sensitivity of the cGAS-STING pathway, thereby promoting the maturation of DCs. Studies have shown that the phosphorylation levels of STING and downstream proteins in the cGAS-STING pathway increase significantly following PTT (237). PTT-induced mitochondrial permeabilization and DNA damage release mtDNA, which activates cGAS and STING (238). STING activates IRF3 for type I interferon (IFN-I) responses and simultaneously activates nuclear factor kappa-light-chain-enhancer of activated B cells (NF-κB), which induces IL-6 production. Moreover, these inflammatory cytokines can lead to NF-κB activation in tumor cells and promote the coexpression of COX-2, therefore driving the tumor EMT process (239). IL-6 binds to its receptor on tumor cells, activating JAK-STAT3 via STAT3 Y705 phosphorylation (240). Phosphorylated STAT3 then binds the PD-L1 promoter, upregulating PD-L1 through an autocrine IL-6 loop (241). This axis has been characterized in TNBC cells, where chromosomal instability and micronucleus formation amplify STING signaling (241). It is reported that STING-negative tumor cells lack this STAT3-PD-L1 response, suggesting that STING expression may serve as a potential biomarker for PTT-ICI responsiveness. ICD induced by PTT can promote the release of DAMPs, further promoting the maturation of DCs (**Figure 7**). At the same time, the activated cGAS-STING pathway promotes the production of type I interferons and other cytokines, promoting the activation and infiltration of CD8^+^ T cells (237). For instance, the PTT therapy administered via intravenous injection of PLGA-PEG-ICG-R837 not only significantly promotes the maturation of DCs but also enhances the secretion of various proinflammatory key cytokines (242). As a key IFN-I molecule, IFN-β can stimulate the maturation and cross-presentation function of DCs, thereby enhancing the T lymphocyte immune response (243). Tumor cells can also inhibit the cGAS-STING signaling pathway by disrupting ER-related metabolism. For instance, in BC, hypoxia induces the translocation of the metabolic enzyme ADSL to the ER (244). The metabolite fumaric acid generated by ADSL binds to STING, blocking the activation of STING by cGAMP and thereby inhibiting the production of cytokines such as IFN-I.

The hyperthermia and ROS produced by PTT target the ER, inducing severe ERS (53, 54). Research has found that ROS produced by PTT immediately oxidize the proteins within the ER, disrupts the calcium ion homeostasis within the ER, and directly attacks the ER membrane (57). This stress response induces the UPR and eventually leads to the exposure of “eat me” signals such as CRT on the surface of tumor cells. CRT can efficiently promote the uptake, processing, and presentation of tumor antigens by DCs, thereby enhancing immune responses (14). Studies have shown that, compared with untargeted nanosystems, ER-targeted nanosystems can induce stronger ER stress and CRT exposure under near-infrared light irradiation, thereby enhancing immune responses (245). During PTT, high concentrations of ROS and inflammatory factors released by dead tumor cells may induce oxidative stress in infiltrating T cells. Li et al. demonstrated through an *in vivo* clearance experiment of CD8^+^/CD4^+^ T cells that the ICD induced by ER-targeted phototherapy depends on T cells (57). However, whether the pressure is sufficient to trigger ERS in T cells still requires further experimental research to confirm or rule out.

Increasing evidence indicates that ERS triggered by chemotherapy, phototherapy, or other factors can induce ICD in tumor cells (246). The latest research shows that local hyperthermia induced by PTT within the ER leads to ERS and can subsequently induce pyroptosis in immunogenic cells. Experimental results show that the pyroptosis induced by PTT is achieved through the GSDME-mediated pathway. The cleaved GSDME-NT can cause cell membrane perforation, which is consistent with previous studies on ERS-induced GSDME-dependent pyroptosis (247). Notably, under hypoxic and normoxic conditions, the pyroptosis induced by PTT follows the same GSDME pathway. This finding is particularly important because hypoxia, as a typical feature of aggressive solid tumors, often weakens the efficacy of traditional therapies (248). The ability of PTT to induce pyroptosis in hypoxic environments provides a new idea for overcoming this limitation and improving the therapeutic effect of solid tumors. Moreover, this pyroptosis triggered by PTT occurs in various cancer cell lines such as HepG2, 4T1, and B16-F10, highlighting the wide applicability of this mechanism.

Collectively, these organelle-specific stress responses, ranging from ER-derived CRT exposure to mitochondria-derived mtDNA release, converge on the activation of innate immune sensors, providing the initial trigger for downstream adaptive immunity.

### 6.2 Metabolic reprogramming of tumor-infiltrating immune cells driven by TME metabolites

Mitochondrial homeostasis in immune cells is disrupted in the TME through nutrient deprivation, accumulation of metabolic waste, and metabolic reprogramming, thereby affecting immune function. For instance, glutamine deprivation in tumor cells leads to a decrease in MMP, an increase in ROS levels, and mitochondrial swelling (249). Such structural damage induces apoptosis, reducing the secretion of effector molecules such as perforin and granzyme B, thereby impairing T-cell cytotoxicity. In addition, citric acid released from the mitochondrial matrix in the TME is converted into acetyl-CoA, which is used for the large-scale synthesis of fatty acids, leading to abnormal accumulation of lipid droplets in the cytoplasm (250). Lipid accumulation potentiates T-cell dysfunction and exhaustion by promoting lipid peroxidation (251). Meanwhile, depletion of methionine significantly reduces the survival rate of CD8^+^ T cells and inhibits the production of IFN-γ and TNF-α, while Met supplementation can effectively reverse these effects. Compared to free Met, MPC@Fe increased *tumor-infiltrating* IFN-γ+CD8^+^ T cells by 4.2-fold under PTT, with a 27.3-fold reduction in tumor volume (220). For instance, lactic acidosis suppresses glycolytic flux and degranulation in CTLs, reducing the secretion of perforin and granzymes (252). Lysosomal acidification driven by V-ATPase is essential for mTORC1 activation, which in turn regulates T-cell differentiation and the generation of memory T cells. Inhibition of V-ATPase assembly mediated by factors, such as Natural Killer Cell Granule Protein 7, compromises T-cell persistence and memory phenotype acquisition (253). Notably, tumor cells utilize lysosomes to promote immune evasion, providing a new target for the combined therapy based on PTT. For instance, tumor cells can induce high expression of lysosome-associated membrane protein Lysosome-associated membrane protein 2a in tumor-associated macrophages (TAMs). By enhancing their lysosomal degradation activity, they specifically degrade proteins such as PRDX1 and CRTX1, thereby promoting the polarization of macrophages toward a protumor M2 phenotype and maintaining an immunosuppressive TME (254). This suggests that interfering with Lysosome-associated membrane protein 2a (LAMP2a) or the protein degradation pathways mediated by LAMP2a may reverse macrophage polarization and synergize with the immune activation effect of PTT.

Lysosomes also mediate T-cell metabolic reprogramming in response to TME-derived signals. V-ATPase-driven lysosomal acidification is essential for mTORC1 activation, which orchestrates T-cell differentiation and memory formation. Disruption of V-ATPase assembly compromises T-cell persistence and memory acquisition via Natural Killer Cell Granule Protein 7 (NKG7) (253). Thus, this metabolic stress shapes the bioenergetic state of tumor-infiltrating lymphocytes through lysosomal pH and mTORC1 signaling. Lysosomal membrane permeation induced by PTT further contributes to DAMP release and NF-κB activation, thereby enhancing immune response (255). However, an inadequate thermal dose during PTT may lead to lysosomal dysfunction, exacerbating local acidosis and ionic imbalance, and further inhibiting the activity and proliferation of effector immune cells such as CTLs and NK cells (179). This dose-dependent duality must be considered when designing a combined treatment plan based on PTT. Tumor cells exploit LAMP2a-mediated chaperone-assisted autophagy in TAMs to promote M2 polarization and immunosuppression (254). Whether PTT modulates this pathway remains unexplored. Lysosomal gene expression signatures such as KCNE1, NPC2, and SFTPD have been correlated with immune infiltration patterns, suggesting their potential as biomarkers for patient stratification in PTT-immunotherapy combinations (256).

### 6.3 Synergistic strategies combining PTT with metabolic-immune checkpoints

PTT-induced ICD releases ATP into the extracellular space, acting as a “find-me” signal that recruits DCs and APCs. However, CD39 and CD73 ectoenzymes hydrolyze ATP to adenosine, an immunosuppressive metabolite that activates A2AR on T and NK cells, suppressing their effector functions (257). Immunosuppressive factors such as adenosine (Ado) further inhibit T-cell function through the A2A receptor (A2AR) signaling pathway, leading to immunosuppression (258). Thus, PTT-induced ATP release is counterbalanced by adenosine-mediated immunosuppression. To address this, Zhao et al. developed targeted nanorods Apt@SCH@BP, which kill tumor cells and remodel the TME through the synergistic effects of phototherapy and A2AR blockade. This approach promotes DCs maturation and CTL activation while inhibiting regulatory T cells and disrupting stromal barriers such as CAFs and collagen (259). A study found that Ado metabolism and A2AR expression are negatively correlated with tissue oxygen levels (260). Alleviating tumor hypoxia is expected to reverse the Ado-A2AR metabolic pathway, further promoting antitumor immune responses. For example, Zeng et al. developed MnO_2_-assisted photosynthetic bacteria biohybrids (MnO_2_@PSB) to enhance tumor photothermal immunotherapy by interfering with the Ado-A2AR metabolic pathway (261). This offers a direction for enhancing antitumor immunotherapy through metabolic pathway modulation. These insights support combining PTT with ICIs (262). PTT directly kills tumor cells and generates antigens, while metabolic stress upregulates PD-L1 on surviving cells, rendering them susceptible to PD-1/PD-L1 blockade. Preclinical models confirm that this combination improves tumor control, increases CD8^+^ T-cell infiltration, and reduces Treg frequencies compared with monotherapy (237). Glycolysis inhibitors, such as 2-deoxy-d-glucose, LDHA inhibitors, and glutaminase inhibitor CB839 also synergize with PTT and ICIs by depleting ATP and reducing lactate, a metabolite that promotes PD-L1 and suppresses CTL function (263). A2AR antagonists, such as AZD4635, block adenosine-mediated suppression and have shown favorable safety profiles in early-phase clinical trials (257). Nanoplatforms codelivering PTAs with A2AR antagonists locally inhibit adenosine signaling with minimal systemic toxicity. This enhances CTL and NK infiltration while reducing MDSC accumulation.

## 7 Challenges and future perspectives

Despite its promise in disrupting tumor metabolism via organelle stress, the clinical translation of PTT is hampered by both biological complexity and technical limitations. A deeper understanding of these challenges from the perspective of organelle biology is essential to guide future breakthroughs.

### 7.1 The challenge of tumor heterogeneity in metabolic regulation induced by PTT

PTT triggers metabolic perturbation through multiple organelle stresses, which is significantly influenced by tumor heterogeneity. The main challenge in clinical translation is that tumor type, stress adaptation networks, and mitochondrial function collectively determine whether PTT effectively suppresses tumor metabolism. A systematic understanding of these factors is crucial for patient stratification and the rational design of combined treatment regimens.

The basal metabolic phenotype of tumor cells significantly influences their sensitivity to PTT. Tumors that rely on high OXPHOS, such as osteosarcoma and certain subtypes of BC, are more susceptible to targeted mitochondrial PTT (125). This is because the rapid depolarization of MMP and the blockage of the ETC can trigger an acute energy collapse. In contrast, tumor cells that rely on glycolysis require combined metabolic inhibition methods. In this case, the metabolic interference effect of PTT alone is limited unless combined with glycolysis inhibitors such as 2-DG and PKM2 modulators. Moreover, in highly secretory or mucinous tumors, the basal ERES levels are elevated, making them more responsive to the ERS amplification induced by PTT and subsequently to ICD. However, ERS may enhance biosynthesis through UPR signals and regulate lysosomal function to eliminate toxic by-products and promote metabolic recovery (264). Therefore, when considering the regulation of tumor metabolism by PTT, it is necessary to take into account the ERS triggered by PTT in different tumors.

Notably, the adaptive plasticity exhibited by tumor cells in response to photothermal stress further exacerbates the interference of heterogeneity on therapeutic efficacy. The expression and activity of HSPs, particularly HSP70 and HSP90, vary significantly among different tumors and are closely related to intracellular ATP levels. In glycolytic tumors, ATP generated by substrate phosphorylation can still maintain the function of HSP chaperone proteins under PTT, thereby repairing denatured metabolic enzymes such as PKM2 and HK2 and maintaining thermoresistance. This explains why the combination of PTT with glycolysis inhibitors can overcome thermoresistance. Moreover, metabolic by-products such as lactate can acidify the extracellular space and activate the pH sensor GPR65 on macrophages, thereby inducing an immunosuppressive M2-like phenotype and weakening the antitumor immune response induced by PTT. Therefore, tumors with a higher glycolytic level not only have resistance to direct thermal damage but also promote the inhibition of the immune environment, requiring the simultaneous targeting of lactate metabolism to restore the immune activation mediated by PTT.

Mitochondrial function is another factor that affects the tumor heterogeneity of the damage effect induced by PTT. The expression abundance of ETC complexes varies in different tumors, which influences the mitochondrial damage caused by PTT. Tumors that rely on high activity of complex I/III are prone to RET under PTT thermal stimulation and generate a large amount of ROS. Differences in the levels of Mn-SOD and GSH within the mitochondrial matrix critically dictate whether equivalent ROS production translates into effective cytotoxicity. Differences in substrate compensation ability determine the metabolic pattern after mitochondrial damage. Under the same hyperthermia, AGS gastric cancer cells can enhance OXPHOS compensation to maintain ATP, while Caco-2 and T3M4 cells do not have this ability (34). Beyond these mitochondrial traits, the nuclear stress response further amplifies this heterogeneity. When PTT triggers nucleolar stress or DNA damage, p53 upregulates the TIGAR gene to inhibit glycolysis and switches the metabolic pathway to the PPP. This process can generate NADPH, thereby counteracting the oxidative stress caused by PTT and may promote cell survival. However, when the PTT intensity is sufficiently high to rapidly deplete ATP, the ATP-dependent HSP repair mechanism fails. In this context, p53 can induce cell apoptosis by upregulating proapoptotic effectors such as PUMA and Bax. In contrast, p53-mutant tumors usually exhibit persistent glycolysis activation, enhanced NADPH regeneration, and impaired apoptosis, weakening the metabolic perturbation induced by PTT. Therefore, a combined strategy including inducing ferroptosis/cuproptosis to bypass p53 and inhibiting the PPP can suppress the metabolism of such tumors.

### 7.2 Translational challenges in PTT-based organelle-targeted nanoplatforms

Although the nanoplatforms listed in **Table 1** demonstrate remarkable preclinical efficacy, their clinical translation is severely limited due to manufacturing complexity. Most of these systems rely on multistep supramolecular assembly or intricate surface functionalization, such as PEGylation, ligand conjugation, and membrane coating (265). However, these procedures are highly sensitive to reaction conditions; even minor deviations can markedly affect particle size distribution, polydispersity index, and drug loading, ultimately compromising batch-to-batch reproducibility. For plasmonic materials like silver triangular nanorhombuses (PVA-SNT), their shape, size, and concentration directly determine the efficiency of photothermal generation (266). Therefore, developing simpler and more repeatable synthesis routes, such as biomineralized magnetic nanoparticles (MagMn) based on biological templates, provides new ideas for solving the problem of batch-to-batch reproducibility (267). Moreover, the photothermal performance of organic dyes such as ICG and IR780 is vulnerable to photobleaching and aggregation-caused quenching under physiological conditions, leading to a significant decline in photothermal conversion efficiency. Even inorganic nanomaterials, such as gold nanorods, undergo morphological reshaping and loss of plasmonic resonance upon repeated laser irradiation.

Beyond the well-recognized limitation of NIR-I window penetration, the physical mechanisms of light interaction with tissues also pose significant challenges. Tissue scattering and absorption by endogenous chromophores, such as hemoglobin and melanin, generate intense spatial temperature gradients within the tumor. The tumor periphery often experiences ablative temperatures, while the hypoxic core remains in a sublethal thermal range (40 °C–43°C), which paradoxically activates prosurvival HSF1/HIF-1α pathways and promotes thermotolerance rather than cell death. Moreover, thermal diffusion into adjacent normal parenchyma is unavoidable. Some studies have proposed that intelligent nanoscale platforms based on viscosity or resistance response signals could be used to achieve real-time temperature feedback, severely limiting their clinical application (268, 269). The signals captured by the intelligent nanoscale platforms mainly reflect the local microenvironment around the accumulation site and cannot accurately monitor the actual thermal dose throughout the entire tumor volume. Consequently, PTT currently lacks a reliable feedback control system for real-time power adjustment, increasing the risk of unintended thermal injury to vital structures such as skin, nerves, or vessels. In contrast to these indirect sensing strategies, imaging-guided thermometry, such as photoacoustic tomography, enables full-field thermal mapping across the tumor, offering a more comprehensive assessment of thermal dose distribution. Nevertheless, as exemplified by the recent examples of armored gold nanostars with photoacoustic computed tomography (AC-GNS/PACT), connecting nanomaterial engineering with advanced imaging modalities represents a promising route to overcome these translational barriers (270).

A critical bottleneck in current PTT nanoplatform evaluations is the lack of long-term (≥ 1 year) biodistribution and clearance data. While biodegradable platforms, such as iron-based nanozymes, zinc peroxide, and poly(lactic-co-glycolic acid) nanoparticles, are designed to be cleared via renal or hepatobiliary routes, their degradation kinetics are highly susceptible to local pH and enzymatic activity. Premature degradation leads to loss of therapeutic effect, while delayed degradation prolongs tissue exposure and exacerbates chronic toxicity (271). For nonbiodegradable or inert materials, such as gold, carbon nanotubes, and silicon-based materials, the liver and spleen often serve as long-term reservoirs. It has been reported that their retention time ranges from 6 to 12 months after administration, which may induce oxidative stress, lysosomal dysfunction, and granuloma formation over extended periods. Alarmingly, most preclinical studies terminate within 30–90 days, leaving a substantial knowledge gap regarding chronic immunotoxicity and carcinogenic potential. Furthermore, the metabolic byproducts of degradable nanocarriers, such as Cu^2+^, Fe^3+^, and Zn^2+^, have been shown to interfere with systemic iron and copper homeostasis, which could disrupt erythropoiesis and neurocognitive function (272, 273). Despite these acknowledged risks, current guidelines of regulatory agencies such as the US Food and Drug Administration (FDA) and the European Medicines Agency (EMA) do not specify any explicit requirements for long-term biopersistence studies of photothermal nanomaterials, leading to significant ambiguity in the assessment of risks and benefits (274). These biosafety profiles must be rigorously established in large-animal models prior to any clinical consideration.

Beyond physicochemical and toxicological barriers, the biological responses triggered by nanotechnology platforms also present unique challenges in transformation. DAMPs and mtDNA can activate the cGAS-STING pathway, leading to sustained IFN-I production and excessive secretion of proinflammatory cytokines such as IL-6 and TNF-α. The proinflammatory cytokines not only increase the risk of developing systemic capillary leakage syndrome and multiple organ damage but also may induce abnormal T-cell exhaustion through chronic antigen stimulation. Moreover, the upregulation of PD-L1 driven by NF-κB and STAT3 forms an immune checkpoint feedback loop, partially counteracting the therapeutic benefit. Meanwhile, single nanoparticles that combine PTAs, metabolic inhibitors, and immune adjuvants currently lack regulatory oversight in terms of quality control, release specifications, and preclinical safety assessment. The surface modifications of the nanoplatforms targeting different organelles, such as mitochondria and ER, are different, and the pharmacokinetic differences are extremely significant. Therefore, defining the active pharmaceutical ingredient (API), establishing release specifications for each component, and evaluating their distinct yet interdependent pharmacokinetics and pharmacodynamics pose unprecedented challenges for investigational new drug (IND) applications. The lack of validated surrogate biomarkers for monitoring PTT-induced immune activation in real-time further complicates dose-escalation and scheduling strategies in early-phase clinical trials.

## 8 Conclusions

In recent years, there have been relatively few clinical studies on PTT for tumors, and the existing research has not shown significant therapeutic efficacy. However, PTT based on organelle metabolism offers a glimmer of hope for immunotherapy. This article reviews the mechanisms by which PTT exerts metabolic intervention on tumor immunity in tumors, including metabolic perturbation induced by organelle stress, the tumor adaptive response, and its impact on tumor immunity. Overall, several limitations of PTT must still be addressed, such as tumor heterogeneity, batch-to-batch reproducibility, long-term biological safety, and regulatory complexity. Nevertheless, PTT-based metabolic immune intervention remains a promising avenue for research aimed at improving therapeutic efficacy. In addition, PTT achieves significant antitumor immune effects by interfering with tumor metabolism through organelles, and it can be combined with other methods to amplify its therapeutic effect on multiple organelles. We expect that the continuous development of organelle-based metabolic immunotherapy and innovative strategies targeting TME remodeling will lead to more effective tumor immunotherapies.

## Glossary

PTT: photothermal therapy
TME: tumor microenvironment
NIR: near-infrared
OXPHOS: oxidative phosphorylation
HSPs: heat shock proteins
TNBC: triple-negative breast cancer
BC: breast cancer
PTAs: photothermal agents
TAAs: tumor-associated antigens
DAMPs: damage-associated molecular patterns
APCs: antigen-presenting cells
ROS: reactive oxygen species
SOD: superoxide dismutase
mtDNA: mitochondrial DNA
FASN: fatty acid synthase
GSH: glutathione
ETC: electron transport chain
MMP: mitochondrial membrane potential
Cyt *c*: cytochrome *c*
OMM: outer mitochondrial membrane
mPTP: mitochondrial permeability transition pore
VDAC: voltage-dependent anion channel
Bcl-2: B-cell lymphoma 2
mPTT: mild photothermal therapy
NK: natural killer
ERS: endoplasmic reticulum stress
eIF2α: eukaryotic translation initiation factor 2α
CHOP: C/EBP homologous protein
ATF6: activating transcription factor 6
UPR: unfolded protein response
GRP78: glucose-regulated protein 78
BiP: binding immunoglobulin protein
IRE1α: inositol-requiring protein 1α
XBP1: X-box binding protein 1
PERK: PKR-like endoplasmic reticulum kinase
PINK1: PTEN-induced putative kinase 1
LC3-II: microtubule-associated protein 1 light chain 3-II
HSF1: heat shock factor 1
JNK: c-Jun N-terminal kinase
KD: knockdown
LDHA: lactate dehydrogenase A
PPP: pentose phosphate pathway
DOX: doxorubicin
PKM2: pyruvate kinase M2
G6PD: glucose-6-phosphate dehydrogenase
siRNA: small interfering RNA
GOx: glucose oxidase
PUFAs: polyunsaturated fatty acids
MT: mitoxantrone
ACLY: ATP citrate lyase
Treg: regulatory T cells
HIF-1α: hypoxia-inducible factor-1α
CTL: cytotoxic T lymphocyte
CAFs: cancer-associated fibroblasts
GDEPT: gene-directed enzyme prodrug therapy
TCA: tricarboxylic acid
V-ATPase: vacuolar-type H^+^-ATPase
CRC: colorectal cancer
NK: natural killer
PD-L1: programmed death ligand-1
ER: endoplasmic reticulum
GA: Golgi apparatus
SDH: succinate dehydrogenase
FH: fumarate hydratase
IDH: isocitrate dehydrogenase
MIT: ion therapy
GLUT1: glucose transporter 1
GLO1: glyoxalase 1
GLO2: glyoxalase 2
HK2: hexokinase 2
MIPs: molecularly imprinted polymers
AMPK: adenosine monophosphate-activated protein kinase
CDT: chemodynamic therapy
ClC-3: chloride channel-3
PHD: prolyl hydroxylase
2-DG: 2-deoxy-{{sc}}d{{/sc}}-glucose
GLS1: glutaminase 1
MCT1: monocarboxylate transporter 1
ASCT2: alanine–serine–cysteine transporter 2
SLC3A2: solute carrier family 3 member 2
ESCRT: endosomal sorting complexes required for transport
GSTs: glutathione *S*-transferases
GPX4: glutathione peroxidase 4
P-gp: P-glycoprotein
TAMs: tumor-associated macrophages
CA IX: carbonic anhydrase IX
CAI: carbonic anhydrase inhibitor
CRT: calreticulin
DCs: dendritic cells
ICD: immunogenic cell death
Ado: adenosine
A2AR: A2A receptor
MGO: methylglyoxal
cGAS: cyclic GMP-AMP synthase
STING: stimulator of interferon genes
IFN-I: type I
ECM: extracellular matrix.
